# Uncovering *de novo* polyamine biosynthesis in the gut microbiome and its alteration in inflammatory bowel disease

**DOI:** 10.1080/19490976.2025.2464225

**Published:** 2025-02-09

**Authors:** Xinwei Li, Xia Xiao, Shengnan Wang, Biyu Wu, Yixuan Zhou, Pan Deng

**Affiliations:** a Jiangsu Key Laboratory of Neuropsychiatric Diseases and College of Pharmaceutical Sciences, Soochow University, Suzhou, Jiangsu, China; b Department of Pharmaceutical Analysis, Soochow University, Suzhou, Jiangsu, China

**Keywords:** Polyamine, gut microbiome, stable isotope, metabolomics, inflammatory bowel disease

## Abstract

Polyamines are important gut microbial metabolites known to affect host physiology, yet the mechanisms behind their microbial production remain incompletely understood. In this study, we developed a stable isotope-resolved metabolomic (SIRM) approach to track polyamine biosynthesis in the gut microbiome. Viable microbial cells were extracted from fresh human and mouse feces and incubated anaerobically with [U-^13^C]-labeled inulin (tracer). Liquid chromatography-high resolution mass spectrometry analysis revealed distinct ^13^C enrichment profiles for spermidine (SPD) and putrescine (PUT), indicating that the arginine-agmatine-SPD pathway contributes to SPD biosynthesis in addition to the well-known spermidine synthase pathway (PUT aminopropylation). Species differences were observed in the ^13^C enrichments of polyamines and related metabolites between the human and mouse microbiome. By analyzing the fecal metabolomics and metatranscriptomic data from an inflammatory bowel disease (IBD) cohort, we found significantly higher polyamine levels in IBD patients compared to healthy controls. Further investigations using single-strain SIRM and *in silico* analyses identified *Bacteroides* spp. as key contributors to polyamine biosynthesis, harboring essential genes for this process and potentially driving the upregulation of polyamines in IBD. Taken together, this study expands our understanding of polyamine biosynthesis in the gut microbiome and will facilitate the development of precision therapies to target polyamine-associated diseases.

## Introduction

Gut microbial metabolites play a critical role in host health and disease. Polyamines, such as putrescine (PUT) and spermidine (SPD), are important metabolites of intestinal bacteria that exert local effects and enter the circulation to affect the functions of host organs.^1^ These organic cations are crucial for cell division and proliferation. Additionally, they bind to the phosphate groups of negatively charged DNA and RNA, contributing to messenger RNA translation and nucleic acid stabilization.^2,3^ Moreover, polyamines act as central regulators in inflammatory and autoimmune diseases.^4^ Therefore, polyamines play a vital role in various physiological processes and diseases.^5,6^

In healthy adults feces, polyamines are present at micromole concentrations, with PUT at approximately 791.2 μM and SPD at 56.8 μM.^7^ In the plasma of healthy individuals, PUT is found at 5.3 μM, while SPD is at 0.6 μM.^8^ Dysregulated polyamine metabolism is closely associated with intestinal diseases, including Inflammatory bowel disease (IBD) and colon cancer.^9–11^ For instance, elevated levels of N-acetylputrescine and N1-acetylspermidine have been observed in the plasma of children with ulcerative colitis.^12^ Polyamines and their metabolites, measured in either urine or serum, have shown potential as biomarkers for colon cancers.^13,14^ Conflicting effects of polyamines on intestinal health have been reported, while some reported anti-inflammatory properties and maintenance of mucosal homeostasis by polyamines,^5,15^ other results showed that polyamines promoted oxidative stress and exacerbated inflammation.^16,17^ In addition, although promising anti-cancer results have been obtained from *in vitro* and preclinical models by targeting the cellular polyamine pathway,^18,19^ clinical trials have generally been disappointing.^20^ This disconnect likely stem from unrevealed compensatory mechanisms that arise when cellular polyamine biosynthesis is inhibited. Therefore, a deeper understanding of the polyamine pathway is needed to effectively target their metabolism for therapeutic benefit in the treatment of intestinal diseases.

Polyamines in the intestinal lumen can originate from either dietary source or be produced by the intestinal microbiota. However, food-derived polyamines are often absorbed before reaching the lower intestine,^21^ making the colon’s polyamines predominantly microbiome-derived.^22,23^ There has been considerable interest in microbial polyamine biosynthesis.^24^ Recent research by Matsumoto’s group explored the role of human gut commensals in polyamine biosynthesis. They identified a hybrid system for PUT production by *Faecalibacterium* and *Escherichia*, where PUT is produced via agmatine catabolism consisting of agmatine deiminase, putrescine carbamoyl transferase and an agmatine-putrescine antiporter.^25^ In another study, they found that PUT was produced from arginine via several extracellular intermediates and catalyzed by enzymes derived from multiple bacterial species.^26^

Although significant progress has been made in understanding the microbial regulation of polyamines, the biosynthetic pathways in gut microbiome remain incompletely characterized. Stable isotope resolved metabolomics (SIRM) is a thriving approach that allows dynamic tracking of individual atoms in metabolites through metabolic networks. We previously reported ^13^C-based SIRM methods to study the dynamic metabolism in mouse gut microbiome.^27,28^ In particular, [U-^13^C]-inulin was used as a tracer which shown to be an effective substrate for the gut microbiome, enabling the holistic tracing of microbial metabolic pathways.

In the present study, we first developed a chemoselective derivatization SIRM technology to analyze the stable-isotope labeled polyamines and related metabolites. Using [U-^13^C]-inulin as a tracer, we tracked the biosynthesis of polyamines in the human and mouse fecal microbiome. To further elucidate the role of the polyamine pathway in IBD, the polyamine levels and functional gene expression of intestinal bacteria in IBD were compared with healthy controls by leveraging the fecal metabolomics and metatranscriptomics database. Additionally, single-strain SIRM and *in silico* analyses were used to explore functional gut microbes. The findings revealed a novel *de novo* SPD biosynthesis pathway in the human gut microbiome, underscoring the importance of polyamine bioanalysis in aligning gut microbial functions to host intestinal health.

## Materials and methods

### Materials

The authentic standards of putrescine (PUT, purity ≥ 99.9%) and spermidine (SPD, purity ≥ 99.7%) were purchased from Tan Mo Quality Inspection Co., Ltd (Beijing, China). Tolbutamide (purity ≥99.0%) was purchased from Aladdin (Shanghai, China). The derivatize reagent *N*-(9-fluorenylmethoxycarbonyloxy)succinimide (Fmoc-OSu, purity ≥ 98.0%) and formic acid (LC/MS grade) were purchased from Sigma-Aldrich (Shanghai, China). Methanol, acetonitrile, and other reagents were analytical grade purchased from Sigma-Aldrich (Shanghai, China).The ultrapure water was purchased from Watsons Company (Guangzhou, China). Gifu Anaerobic Medium (GAM) (HB8518–1), chlorhematin (HB03102, 1 mL), and vitamin K1 (HB0310b) were purchased from Hopebio Co., Ltd (Qingdao, China). Anaerobic glove bags (No. 690325, 27’’W × 30’’L) were purchased from NPS Corporation (Cudahy, WI, USA). Anaerobic gas production bags (C-11, 350 mL) were purchased from MGC (Tokyo, Japan). The oxygen indicator resazurin was purchased from Sangon Biotech Co., Ltd (Shanghai, China). [U-^13^C]-inulin (from chicory, ≥97 atom % ^13^C) was purchased from IsoLife bv (Wageningen, Netherlands), and ^12^C-inulin (from chicory root) was obtained from Acros Organics (Geel, Belgium). Hungate anaerobic incubation tubes (FLS-2626–0002) were provided by FM Scientific (Beijing, China).

### Fecal microbiome separation and anaerobic incubations

Three healthy volunteers (two females and one male), aged between 20 and 30 years old, who had not taken antibiotics in the last three months or probiotics in the last month were recruited for this study. The study protocol was approved by the Human Research Ethics Committee of Soochow University under number 2022–001. A fraction of the fecal sample was collected fresh in its native state in a sterile screw-cap container. The samples were quickly transferred to an anaerobic glove bag to ensure fecal microbiome vitality.

Male C57BL/6 mice (6 weeks old, 20–22 g, *n* = 3) were provided by GemPharmatech Co., Ltd. The animals were given *ad libitum* access to food (D12450J, 10% Kcal from Fat, Research Diets) and water. Animal protocols were approved by the Institutional Animal Care and Use Committee of Soochow University under number 202308A0421. Fresh fecal pellets were collected from each mouse in sterile microcentrifuge tubes and quickly transferred to anaerobic bags to ensure microbial activity.

Fresh fecal microbes were individually isolated from three healthy human subjects and three mice, then incubated separately with [U-^13^C]-inulin. Fecal microbial cells were collected as we reported previously.^27^ Briefly, the fresh fecal samples were dissolved in the culture media and processed with a glass rods to suspend the microorganisms and particles. Then, the suspensions were transferred to an anaerobic tube and subjected to low-speed centrifugation (35 *× g*, 10 min) to remove larger particles of undigested material. The supernatants were then collected and centrifuged at 865 *× g* for 10 min to pellet the microbes. After washing the precipitated microorganisms using culture medium, the sediments were collected by centrifugation at 865 *× g* for 10 min. The microbial cells were then suspended in culture medium. [U-^13^C]-inulin or ^12^C-inulin (control group) was added to each tube aseptically to achieve a final concentration of 2 g/L inulin. After incubating at 37°C for 24 h, the samples were centrifuged (865 *× g*, 10 min) to collect the supernatant (culture medium sample). Each pellet was washed with fresh culture medium and centrifuged to collect the microbial cells for further analysis.

### Extraction and derivatization of polyamines for ^13^C enrichment analysis

For the analysis of polyamines, the microbial cells were quenched immediately after collection using 200 μL of acetonitrile containing 0.2% formic acid. The samples were then vortexed and centrifuged, and the resulting supernatant was collected in a microcentrifuge tube as cell extracts. The culture medium from each sample was lyophilized, and the dried powder was dissolved in 200 μL acetonitrile containing 0.2% formic acid for subsequent derivatization. To each 50 μL aliquot of the sample solution, 50 μL of carbonic acid buffer (0.5 M, pH 10.2) was added, followed by 50 μL of 5 mM Fmoc-OSu solution in acetonitrile. The mixture was shaken and incubated at room temperature for 15 min. The derivatization reaction was quenched with 20 μL of formic acid, and the sample was extracted with 500 μL of ethyl acetate. After vortexing for 2 min, the sample was centrifuged and the organic phase was collected and dried under a nitrogen stream. The samples were stored at − 80°C and dissolved in acetonitrile: water (9:1, v/v) before analysis.

For the untargeted metabolomic analysis, the microbial cells were quenched using 450 μL cold methanol immediately after collection. The samples were extracted as previously reported.^27^ Briefly, 5 mL of methyl tert-butyl ether was added to each tube, and phase separation was induced by adding 1.25 mL of deionized water. The samples were then vortexed briefly and centrifuged. Polar fractions were collected into clean tubes and lyophilized. The dried powder was stored at − 80°C and dissolved in methanol: water (8:2, v/v) before analysis.

### SIRM profiling using liquid chromatography-high resolution mass spectrometry (LC-HRMS)

SIRM analyses were performed using either a Q-Exactive HF or a Q-Exactive Focus orbitrap mass spectrometer, both equipped with an Ion Max API source and a HESI II probe, and were coupled to a Dionex UltiMate 3000 UHPLC system (Thermo Fisher Scientific).

The polyamine analysis (intracellular and extracellular polyamine) was achieved using an Agela Technologies Venusil XBP C18 column (2.1 × 50 mm, 5 μm). Mobile phase A was 0.1% formic acid in water, and mobile phase B was acetonitrile. The chromatographic gradient was run at a flow rate of 200 μL/min as follows: 0–10 min, linear gradient from 10% to 90% B; 10–14 min, hold at 90% B; 14–14.5 min, linear gradient to 10% B; maintained at 10% B to re-equilibrate the column. The high resolution mass spectrometer was operated in positive mode with the spray voltage set to 4.0 kV, the heated capillary was held at 350°C, and the HESI probe was set at 325°C. The sheath gas flow was set as 45 units, the auxiliary gas flow was set as 10 units, and the sweep gas flow rate was set to 1 unit. The MS data acquisition was performed in the range of 75–1000 m/z, with the resolution set at 60,000, the AGC targeted at 1e,^6^ and the maximum injection time was set at 200 ms.

Polar extracts (intracellular metabolites) were analyzed by using a SeQuant ZIC-pHILIC column (2.1 × 150 mm, 5 μm). The mobile phases consisted of 0.1% ammonium hydroxide in water with 20 mM ammonium carbonate (A) and acetonitrile (B). The chromatographic flow rate was 150 μL/min. The samples were eluted with a linear gradient from 80% B to 20% B over 20 min, maintained at 20% B for 1 min, increased from 20% B to 80% B for 1 min and hold at 80% B for 6 min. The sample injection volume was 10 μL. The mass spectrometer was operated in positive and negative ionization modes. The MS data were acquired in the mass range of 59–880 m/z, with the resolution set at 70,000.

### Bacterial incubation and SIRM experiment

*B. fragilis* (ATCC25285, bio-53038) and *B. thetaiotaomicron* (ATCC29148, bio-78496) were purchased from BIOBW Co., Ltd (Beijing, China). All procedures were performed under anaerobic conditions. The bacteria were inoculated on a GAM solid medium plate and incubated at 37°C for 48 hours. A single colony was selected and incubated in liquid GAM medium in a Hungate tube at 37°C with shaking. After the OD_600_ reached 0.6, the bacteria were inoculated into a new GAM liquid medium. For the quantification of polyamines in *Bacteroides*, the culture medium and cell pellet were collected at 2 h, 4 h, 6 h, 12 h, 24 h, and 48 h after incubation. After centrifugation at 865 *× g* for 10 min, the cells were quenched and harvested using 200 μL of acetonitrile containing 0.2% formic acid. The supernatant was then derivatized using Fmoc-OSu derivatization as aforementioned, and the organic phase was dried with the nitrogen steam. The sample was reconstituted in 100 μL of acetonitrile containing 500 nM tolbutamide (internal standard), and injected into the liquid chromatography-tandem mass spectrometry (LC-MS/MS) for the quantitative analysis of PUT and SPD. The protein precipitate was washed with acetone and extracted using 0.1% SDS as reported previously.^29^ Protein contents were analyzed using a BCA protein assay kit (Beyotime Biotech, Inc., Shanghai, China).

In the SIRM study, *Bacteroides* spp. were cultured at 37°C until reaching OD_600_ of 0.6. The samples were centrifuged at 865 *× g* for 10 min to pellet the microbes. After washing using the culture medium, the microbial cells were resuspended in clean culture medium. [U-^13^C]- or ^12^C-inulin (non-labeled) were added to each tube (*n* = 3) to achieve a final concentration of 2 g/L. After incubating at 37°C for 24 h, the samples were centrifuged (865 *× g*, 10 min) to collect the supernatant (culture medium sample). The pellet was washed with 1 mL of culture medium and centrifuged to collect the microbial cells, which were then quenched and derivatized using Fmoc-OSu as aforementioned. Samples were stored at − 80°C and dissolved in acetonitrile: water (9:1, v/v) before the LC-HRMS analysis.

### Quantitative analysis of PUT and SPD in Bacteroides using LC-MS/MS

The quantitative analysis of PUT and SPD was performed using an AB SCIEX 4000 QTrap mass spectrometer, equipped with an electrospray ionization source, which was coupled to a Shimadzu LC-20 Liquid chromatography system. Polyamines were separated on an Agela Venusil XBP C18 column (2.1 × 50 mm, 5 μm), and the column temperature was maintained at 40°C. The mobile phase was consisted of water containing 0.1% formic acid (A) and acetonitrile (B). The chromatographic gradient was delivered at a flow rate of 400 μL/min as follows: 0–1 min, linear gradient from 10% to 90% B; 1–3 min, hold at 90% B; 3.1–5 min, hold at 10% B. The sample injection volume was 10 μL. The mass spectrometer was operated in positive modes with the ionspray voltage set to 4500 V, and the ion source temperature held at 450°C. The curtain gas was set to 10 psi, the collision gas was 6 psi, and the ion source gas was 30 psi.

The LC-MS/MS data collection and analysis was performed using Analyst 1.6.3 (AB SCIEX, USA). Calibration curves were constructed by analyzing PUT and SPD spiked calibration samples, which were quantified using the ratio of the LC-MS/MS peak area of the analyte to that of tolbutamide (internal standard). Peak area ratios were plotted against analyte concentrations, and standard curves were constructed using weighted (1/x^2^) least squares linear regression.

### Cohort of patients with IBD and omics data processing

Metabolomics and metatranscriptomics data of patients with IBD and non-IBD controls were collected from the Inflammatory Bowel Disease Multi-omics Database (IBDMDB, https://ibdmdb.org/). Details of the cohort and sampling were described previously by Lloyd-Price et al.^30^ Participants without a diagnosis of IBD were classified as non-IBD controls (healthy volunteers), and subjects with ulcerative colitis or Crohn’s disease were classified as IBD patients. The omic samples were selected using a systematic randomization process. First, the IBDMDB was screened to exclude samples from patients who had taken antibiotics and/or immunosuppressants within two-weeks before sampling. Next, the Excel’s random function was applied to randomly select 400 samples from this filtered dataset. Then, the availability of metatranscriptomic data for each sample was manually verified. Some patient entries lacked corresponding metatranscriptomic data and they were removed from further analysis, resulting in a final set of 375 samples, which were use in the metatranscriptomic analysis. After manually screening the dataset from each sample ID to confirm that the questionnaire was completed and the corresponding metabolomic data were also available, a total of 270 samples were finally used in the metabolomic study, including 135 healthy volunteers and 135 IBD patients. Detailed information of subjects is summarized in Table S1 and S2.

For the analysis of polyamines and related metabolites, human fecal metabolomics data collected using LC-HRMS were downloaded from the IBDMDB. According to the sample processing protocol within this database,^30^ the stool samples were mixed with 100% ethanol, and after centrifugation to pellet the stool, the supernatant was used for metabolomic analysis. Therefore, the polyamine data reflect a combination of metabolites derived from both the gut microbiome and the intestinal lumen. The LC-HRMS peak areas of seven biochemicals, including putrescine, N-acetylputrescine, spermidine, N1-acetylspermidine, N1,N12-diacetylspermine, N1-acetylspermine, and agmatine in these human fecal samples were compared between the IBD patients and controls.

For the investigation of proteins involved in polyamine synthesis in human gut microbes, enzymes related to SPD metabolism were queried in the NCBI RefSeq database, including TTHA1129, TTHA0824, speA, speC, speE and arginase, and the accession numbers were used for BLASTP analysis. Sequences with query coverage ≥ 45% and e-value ≤1 × 10^−5^ were included in the analysis. Protein name and enzyme commission (EC) number were retrieved from KEGG (https://www.kegg.jp/). The transcription level of those enzymes in gut microbiome were subsequently obtained from the IBDMDB based on the EC numbers and exported to Excel (Microsoft Office). The metatranscriptomic data in IBDMDB were analyzed to identify the linkages between enzymes and microbial genera, and the Sankey diagram was created to visualize the linkages, which was drawn using RAWGraphs 2.0 (https://app.rawgraphs.io/).^31^

### Phylogenetic analysis of bacterial polyamine synthesis genes

Translated BLAST (protein sequence searched against translated nucleotide sequences) was used to identify microbial species with genes encoding polyamine metabolism enzymes in the NCBI Prokaryotic RefSeq Genomes database.^32,33^ This tool works by translating nucleotide sequences from the database into hypothetical amino acid sequences.^34^ The intestinal bacteria species were limited to the top 100 as hits for organisms with an e-value threshold of 1e^−5^ and a cutoff of 40% for query coverage. This query cutoff value was chosen based on methodologies from previous studies. For instance, Chittim et al. used a cutoff of 30% in their BLASTP analysis to investigate the distribution of phospholipase D enzymes in commensal gut bacteria.^35^ Qin et al. applied query coverage thresholds of 40% or 45% to identify gut microbial enzymes involved in 2-hydroxybutyric acid synthesis.^36^ In another study, Song et al. set a higher cutoff of 62% when analyzing bile salt hydrolases in human gut microbes.^37^ We selected a medium cutoff of 40% to balance specificity and sensitivity – minimizing false positives while ensuring the inclusion of potential homologs relevant to our analysis. Retrieved sequences were aligned with Multiple Alignment using Fast Fourier Transform (MAFFT, Version 7.520) and analyzed by IQ-Tree v 2.2.2,^38,39^ a fast search algorithm to infer phylogenetic trees by using the maximum likelihood (MLE) method. The ultra-fast Bootstrap (UFBoot) method was then used to evaluate the clade support of phylogenetic trees,^40^ and the best model was automatically selected by using the ModelFinder function. The resulting phylogenetic tree was formatted by ETE3,^41^ and further cosmetic optimizations were performed with ChiPlot (https://www.chiplot.online/).^42,43^

### Data processing, statistics, and visualization

Polar metabolites were identified by comparison of the ion features in the samples with an in-house reference library of authentic chemical standards that included retention time, precursor ions, and their associated product ion mass spectra as we reported previously.^28^ The peak areas of the metabolites and their isotopologues were integrated and exported to Excel via the Thermo Scientific Xcalibur (version 4.2.47). Fractional ^13^C metabolite enrichment was obtained after natural abundance stripping using Escher-Trace (https://escher-trace.github.io/app/index.html.), and the ^13^C fraction of each isotopologue in metabolite was calculated using the protocol described previously.^27,44^ Statistical analyses of the metabolite data and correlation analysis between polyamines and gut microbe genes were performed using the GraphPad Prism version 8.01 for Windows (GraphPad Software Inc., La Jolla, CA, USA). A Student’s t test was applied for paired groups, while the Mann-Whitney test was applied for the unpaired group analysis of IBD datasets. A p-value of less than 0.05 was considered statistically significant for all analyses.

## Results

### Derivatization of polyamines and related metabolites

The derivatization process was first optimized by adjusting the solvent, derivatization reaction time (5, 15, and 25 min) and buffer pH (8.2, 9.2 and 10.2). Acetonitrile containing 0.2% formic acid was used as the solvent for deproteinization, as the acidic environment effectively facilitated the separation of polyamines from other endogenous substances in the biological matrix. While previous studies have used alkaline borate buffer for Fmoc-OSu derivatization,^45^ borate’s tendency to form stable complexes with hydroxyl groups can interfere with pH stability.^46^ Therefore, carbonate buffer was employed in this study. The optimal derivatization reaction yield was obtained by adjusting the pH to 10.2 with a reaction time of 15 minutes (Figure S1).

**Figure 1.**
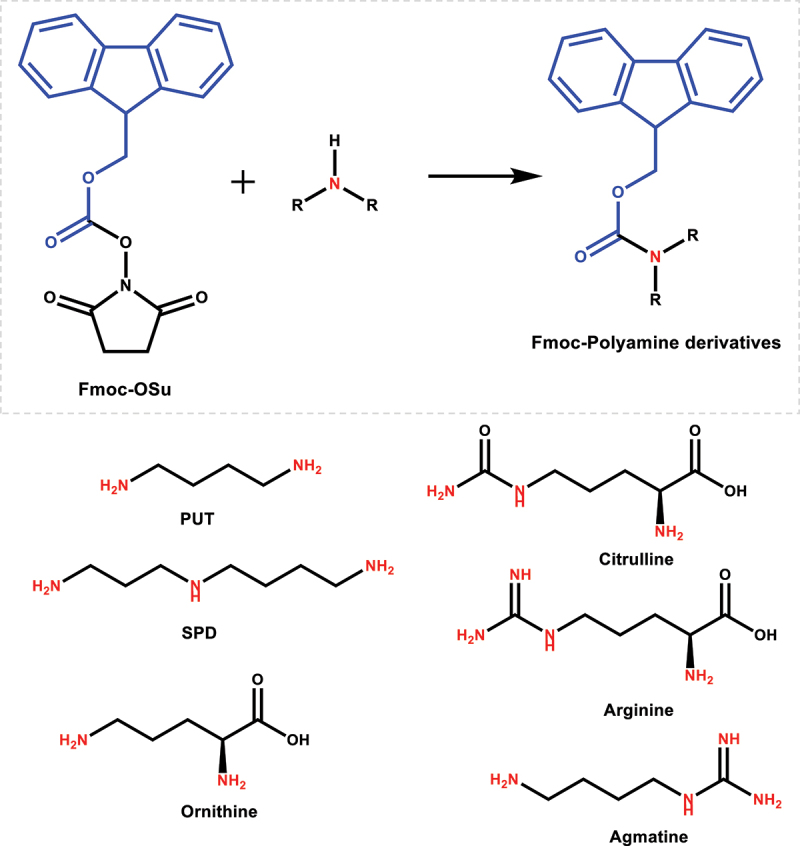
Fmoc-OSu derivatization reaction of polyamines (PUT and SPD) and related metabolites (citrulline, arginine, ornithine, and agmatine). The primary and secondary amino groups that could react with fmoc-OSu are highlighted in red.

**Figure 2.**
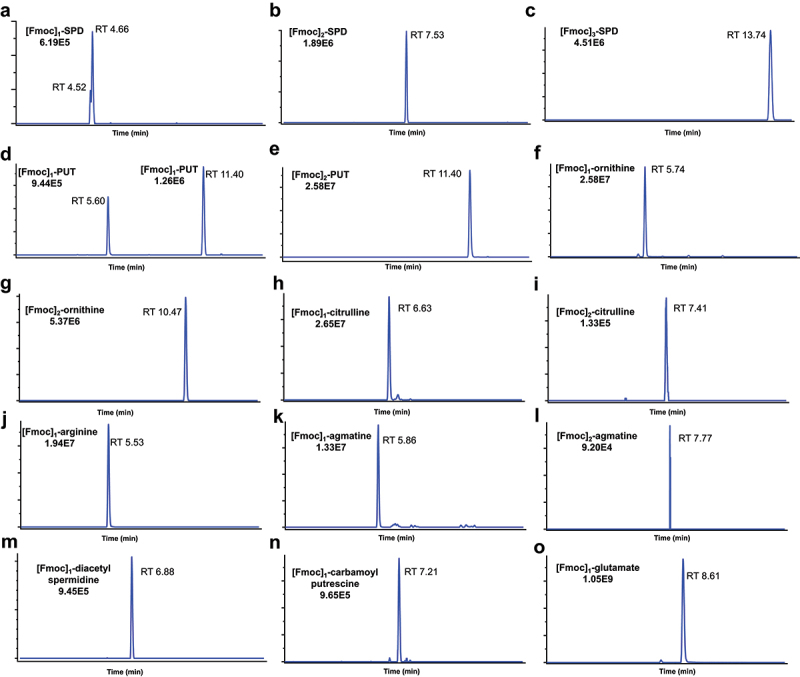
Extracted ion chromatograms of polyamines and related metabolites detected by LC-HRMS after fmoc-OSu derivatization. The derivatized products with the highest LC-HRMS response were used for SIRM analysis. (a) [Fmoc]_1_-SPD; (b) [Fmoc]_2_-SPD; (c) [Fmoc]_3_-SPD; (d) [Fmoc]_1_-PUT; (e) [Fmoc]_2_-PUT; (f) [Fmoc]_1_-ornithine; (g) [Fmoc]_2_-ornithine; (h) [Fmoc]_1_-citrulline; (i) [Fmoc]_2_-citrulline; (j) [Fmoc]_1_-arginine; (k) [Fmoc]_1_-agmatine; (l) [Fmoc]_2_-agmatine; (m) [Fmoc]_1_-diacetylspermidine; (n) [Fmoc]_1_-carbamoylputrescine; (o) [Fmoc]_1_-glutamate.

Polyamines are small polycationic molecules that are challenging to analysis directly using the classical LC-MS approaches. Chemoselective derivatization is a promising method in the stable isotope metabolomics analysis which can enhance MS detection by introducing a spectrally discernable tag on a particular functional group.^47,48^ In the current study, Fmoc-OSu was used as the derivatizing reagent (Figure 1), which can selectively react with amino groups to form stable derivatives with improved LC-MS detection sensitivity. Due to the presence of primary and secondary amino groups in polyamines, Fmoc-Osu can react with both amino groups and lead to multiple Fmoc-derivatized products. For SPD, co-eluted peaks were observed at 4.52 min and 4.66 min in the extracted ion chromatogram of mono-Fmoc derivatized product (Figure 2a), suggesting the presence of [Fmoc]_1_-SPD isomers. The highest LC-HRMS response was observed for [Fmoc]_3_-SPD among the three derivatized products (Figure 2a–c), therefore the fully derivatized SPD ([Fmoc]_3_-SPD) was used in further SIRM analysis. Similarly, two LC peaks were observed for [Fmoc]_1_-PUT (Figure 2d), corresponding for the two mono-Fmoc derivatized products. The fully derivatized product ([Fmoc]_2_-PUT) was used for further SIRM analysis due to its higher LC-HRMS signal intensity (Figure 2e).

Ornithine, citrulline, arginine, agmatine, diacetylspermidine, carbamoylputrescine and glutamate are biochemicals related with polyamine metabolism. The current analysis revealed that mono-Fmoc derivatives were the predominant species for these metabolites (Figure 2f-o), which were subsequently used in the SIRM analysis. Detailed information of Fmoc-derivatized products is provided in Table S3.

### SIRM analysis of metabolites in microbial cells

**Figure 3.**
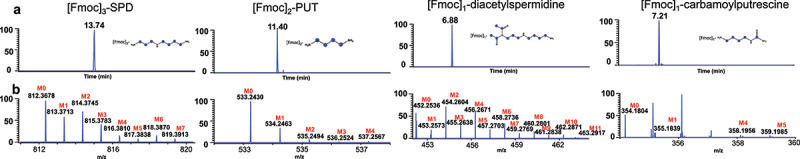
LC-HRMS analysis of fmoc-OSu derivatized polyamines, including [Fmoc]_3_-SPD, [Fmoc]_2_-PUT, [Fmoc]_1_-diacetylspermidine, and [Fmoc]_1_-carbamoylputrescine, in human fecal microbial cells after incubation with [U-^13^C]-inulin. Panel (a) extracted ion chromatogram of fmoc-derivatized polyamines. Panel (b) full scan MS spectra of fmoc-derivatized polyamines in the sample which show the ^13^C labeling profiles.

**Figure 4.**
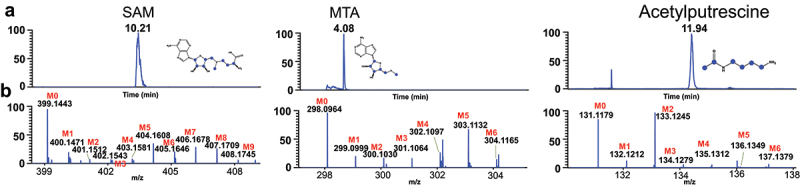
Examples of metabolites detected by HILIC-HRMS in human fecal microbial cells incubated with [U-^13^C]-inulin. Panel (a) extracted ion chromatogram of SAM, MTA, and acetylputrescine. Panel (b) full scan MS spectra of the metabolites which show the ^13^C labeling profiles.

Stable isotope labeling of polyamines and related metabolites were first analyzed in the human fecal microbial cells isolated from three healthy individuals which were separately incubated with [U-^13^C]-inulin. As shown in Figure 3, ^13^C_1_ to ^13^C_n_ (*n* = the total carbon number in a biochemical) isotopologues were detected for [Fmoc]_3_-SPD, [Fmoc]_2_-PUT, [Fmoc]_1_- diacetylspermidine, and [Fmoc]_1_-carbamoylputrescine. Then, HILIC-HRMS method was used for the global analysis of polar metabolites in the microbial cells as we reported previously.^27,28^ A total number of 63 ^13^C-labeled metabolites were detected (Table S4), which were mainly enriched into central carbon metabolism and amino acid metabolism (Figure S2). Methionine metabolism produces the universal methyl donor S-adenosyl-methionine (SAM), which is an aminopropyl donor in the polyamine biosynthesis. Further metabolism of SAM by S-adenosylmethionine decarboxylase produces 5-methyl-adenosine (MTA). Isotopologues of ^13^C_1_ to ^13^C_9_ were detected for SAM, and isotopologues of ^13^C_1_ to ^13^C_6_ were found for MTA, with the major isotopologues being ^13^C_5_ species for both biochemicals, corresponding to the ^13^C labeling of ribose (Figure 4). In addition, acetylputrescine was detected using HILIC-HRMS with ^13^C_2_ being the major isotopologue (Figure 4b). These results suggested that combining Fmoc-derivatization-LC-HRMS with HILIC-HRMS enabled broad detection coverage of stable isotope-labeled metabolites involved in polyamine pathways.

**Figure 5.**
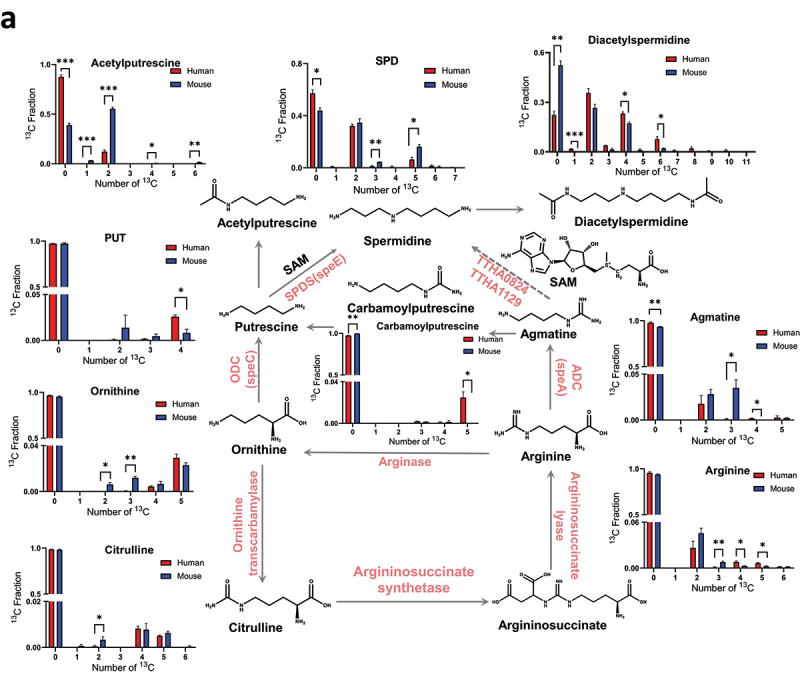
Proposed biosynthetic pathway of polyamines and ^13^C fractional enrichment of metabolites in the human and mouse fecal microbiome incubated with [U-^13^C]-inulin. (a) metabolites detected in the cellular fractions. (b) metabolites detected in culture media. Fresh fecal microbes were individually isolated from three healthy human subjects and three mice, then incubated separately with [U-^13^C]-inulin. The samples were analyzed by LC-HRMS as described in the materials and methods. The ^13^C-carbons were traced through the ornithine-PUT-SPD and arginine-agmatine-spd pathways. The x-axis in the bar chart denotes the number of ^13^C atoms present in each metabolite, and the y-axis indicates the ^13^C fractional enrichment. Values shown are mean ± SEM (*n* = 3). *p < 0.05; **p < 0.01; ***p < 0.001 as indicated.

**Figure 5.**
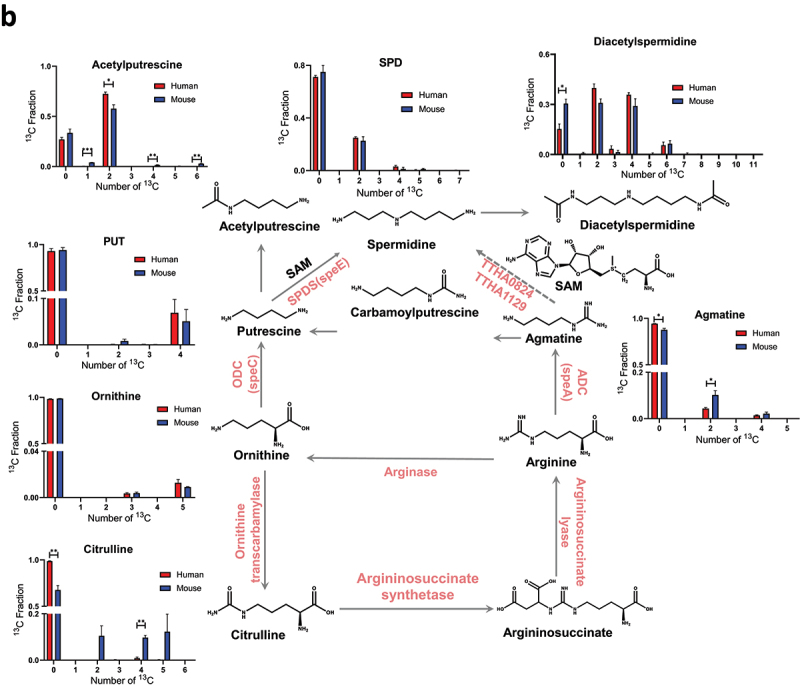
(Continued).

### Agmatine contributes to SPD biosynthesis in the human gut microbiome

PUT is an important precursor for SPD biosynthesis in the microbes. Arginase first cleaves arginine to form ornithine, which is then decarboxylated by ornithine decarboxylase (ODC) to produce putrescine. Finally, spermidine synthase (SPDS) converts PUT to SPD (Figure 5a). The ^13^C fractional enrichment analysis of amino acids in polyamine pathways revealed multiple isotopologues for arginine, ranging from ^13^C_2_ to ^13^C_6_, with ^13^C_2_ being the major labeled species, accounting for 2.6% of the total carbon pool (∑C). Tracking ^13^C metabolism through the ornithine-PUT-SPD pathway, it was found that the major isotopologue for ornithine was the fully labeled ^13^C_5_ species, accounting for 2.9% of ∑C, followed by the ^13^C_4_ species, which accounted for 0.4% of ∑C. Consistent with this labeling pattern, ^13^C_4_ and ^13^C_5_ isotopologues were detected for citrulline at 0.8% and 0.5% of ∑C, respectively, formed by the carbamoylation of ornithine. Decarboxylation of ornithine produced PUT, with ^13^C_2_ to ^13^C_4_ isotopologues of [Fmoc]_2_-PUT being detected, where the fully labeled species (^13^C_4_) was the major ^13^C isotopologue accounting for 2.6% of ∑C, similar to the labeling pattern of ornithine. Further aminopropylation of PUT generated SPD; however, the ^13^C fractional enrichment of ^13^C_4_-SPD was significantly lower than that of ^13^C_4_-PUT, accounting for only 0.6% of ∑C. In contrast, ^13^C_2_ was the major labeled species for SPD, representing 32.2% of ∑C. The major Isotopologue of diacetylspermidine were ^13^C_2_ and ^13^C_4_, accounting for 35.6% and 23.1% of ∑C, respectively, which may derive from the ^13^C_2_-SPD. Notably, the ^13^C_2_ isotopologues of PUT accounted for only 0.07% of the ∑C, which was significantly lower than the ^13^C_2_ labeling of SPD. Michael’s group previously reported that agmatine can be converted into PUT through the production of carbamoylputrescine in plant, after which PUT serves as a precursor for the synthesis of SPD.^49^ The current SIRM analysis revealed that the major isotopologue of carbamoylputrescine was ^13^C_5_, accounting for 2.5% of the ∑C. This labeling pattern was similar to that of PUT but significantly different from that of SPD. Taken together, these findings suggested that alternative pathways contributed to the biosynthesis of SPD besides PUT aminopropylation.

Arginine decarboxylase (ADC; the speA product) catalyzes the transformation of arginine to agmatine. It was reported that homologs of ADC are highly conserved in Bacteroides and Parabacteroides species.^50^ The arginine-agmatine-SPD pathway has been reported as an alternative route for the biosynthesis of SPD in thermophilic bacterium (*Thermus thermophilus HB8*)^51^ and archaea (*Thermococcus kodakarensis KOD*1).^52^ However, this pathway has not been detected in human gut commensals. In this pathway, arginine is metabolized by arginine decarboxylase (ADC) to produce agmatine, which is then converted into SPD through a series of steps involving agmatine aminopropyltransferase and N1-aminopropylagmatinase ureohydrolase. SIRM analysis revealed multiple isotopologues of agmatine, ranging from ^13^C_2_ to ^13^C_5_. Notably, the predominant species was ^13^C_2_-agmatine, similar to that observed for SPD (Figure 5). This suggested that agmatine may provide the ^13^C_2_-labeled precursor for the biosynthesis of ^13^C_2_-SPD, and could therefore directly contribute to the SPD biosynthesis in the human gut microbiome (Figure 5).

In order to understand the exchange of polyamines between intracellular and extracellular pools, metabolites in microbial culture media were analyzed using the Fmoc-OSu derivatization method (Figure 5b). We found that in microbe cells and culture media, similar labeling patterns were observed for polyamines and related metabolites. However, there are differences in the extent of ^13^C fractional enrichment (Figure S3), indicating the potential contributions of transporters to the intra- and extra-cellular pool of metabolites. These findings suggested that the newly synthesized polyamines in microbe cells could be released into the extracellular pool.

### Comparison of polyamine biosynthesis in the mouse and human fecal microbiome

Mouse models have been widely used to investigate the role of polyamines in pathological conditions.^53,54^ Therefore, we employed SIRM to compare fecal microbial catalyzed polyamine biosynthesis between the mice and humans. The ^13^C labeling of metabolites in mouse and human fecal cells are shown in Figure 5. In both mouse and human samples, the predominant isotopologue of SPD was ^13^C_2_, with comparable fractional enrichment (34.7% in mice and 32.2% in humans). However, the enrichment of ^13^C_3_-SPD and ^13^C_5_-SPD was significantly greater in the mice than in the human samples (4.5% vs. 1.1% and 16.2% vs. 6.4%, respectively). This can be partially explained by the higher fractional enrichment of ^13^C_3_-agmatine in the mouse samples. For putrescine (PUT), the major isotopologues were ^13^C_3_ and ^13^C_4_ in mice, whereas ^13^C_4_ predominated in humans. The enrichment of ^13^C_4_-PUT was lower in mice (0.8% vs. 2.6%), consistent with the lower enrichment of ^13^C_5_-carbamoylputrescine in the mouse samples. In both species, ^13^C_5_ was the dominant isotopologue for ornithine. However, mice showed higher enrichment of ^13^C_2_ and ^13^C_3_ isotopologues, corresponding with the increased fractional enrichment of ^13^C_2_-citrulline. These species-specific differences in labeling patterns highlight distinct metabolic pathways and potential variations in microbial functions between mice and humans.

### Bacteroides contributes to polyamine biosynthesis in IBD patients

**Figure 6.**
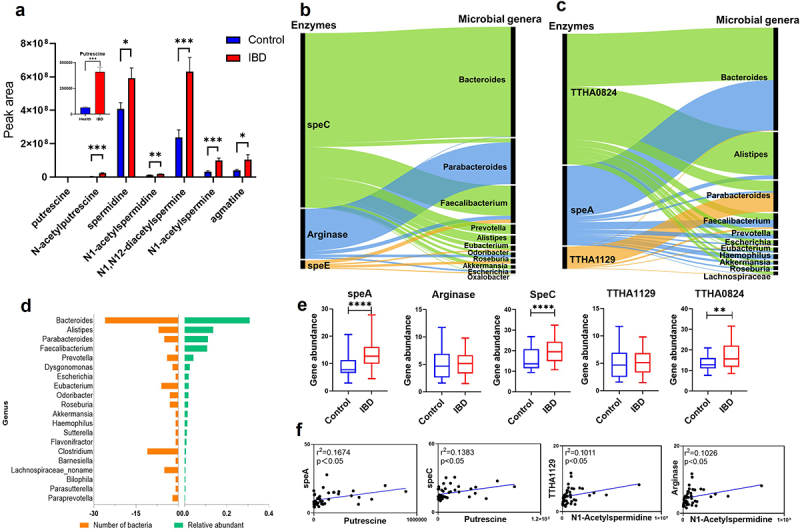
Analysis of polyamine pathways using the public available fecal metabolomic and metatranscriptomic data of IBD patients (https://ibdmdb.Org/). (a) higher levels of polyamines in IBD patients than in control subjects were observed by analyzing the metabolomic data. (b) Sankey diagram displays linkages between enzymes and gut microbiome genera in the ornithine-PUT-SPD pathway. (c) Sankey diagram displays linkages between enzymes and gut microbiome genera in the arginine-agmatine-spd pathway. (d) the biochemical potential of the gut microbiome in the HMP database was examined for the capability to produce polyamines. Shown are the top 20 genera with the highest number of bacteria involved in polyamine biosynthesis. Panel (e) different abundances of microbial genes coding enzymes for polyamine biosynthesis between IBD patients and controls. Panel (f) correlations between polyamines and microbial genes. *p < 0.05; **p < 0.01; ***p < 0.001; ****p < 0.0001 as indicated.

To further explore the bacteria that are responsible for polyamine biosynthesis, and the implications of the pathway in the context of human pathological conditions, the polyamine metabolism was analyzed by leveraging the available omics data of IBDMDB. The differences in fecal polyamine metabolites were compared between IBD and healthy individuals. The demographic information on the 135 controls and 135 IBD patients was shown in Table S1 and IBD-specific information was shown in Table S2, including ulcerative colitis vs. Crohn’s disease breakdown, Montreal classification, Simple Clinical Colitis Activity Index (SCCAI), Harvey-Bradshaw index (HBI), fecal calprotectin and extraintestinal manifestations. Analysis of polyamines and related metabolites, including putrescine, N-acetylputrescine, spermidine, N1-acetylspermidine, N1,N12-diacetylspermine, N1-acetylspermine, and agmatine, showed that the levels of those metabolites in fecal samples from IBD patients were significantly higher than those from healthy subjects (Figure 6a). We further analyzed polyamine levels across different disease states using fecal calprotectin as a biomarker (Figure S4). In ulcerative colitis patients, we observed a significant increase in putrescine levels with rising fecal calprotectin levels, along with similar trends in N-acetylputrescine and N1, N12-diacetylspermine, though these were not statistically significant. In contrast, no significant changes in polyamine levels were observed in Crohn’s disease patients across varying fecal calprotectin levels (Figure S4). These findings suggested potential differences in polyamine dynamics between ulcerative colitis and Crohn’s disease, warranting further investigation to explore causal relationships between polyamines and disease stage.

Metatranscriptomics data were subsequently analyzed to identify the major polyamine-producing species in human gut microbiome. Proteins with sequences closely related to polyamine metabolism enzymes were identified in the RefSeq Select proteins Database (Table S5). Using the EC number of these proteins, metatranscriptomics data of 375 samples were retrieved for analysis. A Sankey diagram was generated to show the associations between these enzymes and gut microbial genera (Figure 6b,c), with the different colored curves indicate the specific microbial genera to which each enzyme belongs. Sugiyama et al. found that intestinal bacteria may have a variety of proteins that were involved in the uptake or export of polyamines.^50^ Information on these transporters is summarized in Table S6. BLAST analysis was performed on these transporters, and a Sankey diagram was drawn to show the connections between the transporter and the gut microbiome (Figure S5).

For the ornithine-PUT-SPD pathway (Figure 6b), *Bacteroides* exhibited the highest speC gene abundance, followed by *Faecalibacterium*, *Prevotella*, and *Alistipes*. Moreover, *Parabacteroides* showed the highest gene abundance of arginase, followed by *Faecalibacterium*, *Odoribacter*, and *Escherichia*. Figure 6c shows the gene abundance of enzymes in the arginine-agmatine-SPD pathway, where *Bacteroides* showed the highest gene abundance of TTHA0824, followed by *Alistipes*, *Parabacteroides*, and *Faecalibacterium*. The highest gene abundance of speA was observed in *Bacteroides*, followed by *Haemophilus*, *Prevotella*, and *Faecalibacterium*. Notably, although *Alistipes* expresses a high gene abundance of TTHA0824, it hardly expresses TTHA1129. Interestingly, *Bacteroides, Parabacteroides*, and *Faecalibacterium* express all three enzymes in this metabolic pathway, suggesting their potential to independently produce SPD. Collectively, these findings suggested that different enzyme expressions in the gut microbiome might be associated with the varying levels of polyamines observed in human feces. Additionally, the enzymes involved in polyamine biosynthesis are widely distributed among gut microbes, with *Bacteroides* emerging as a major genus, considering both the number of bacteria stains within the genus and its relative abundance in the human gut (Figure 6d).

To compare the genes coding polyamine biosynthesis enzymes in gut microbes between IBD patients and healthy individuals, metatranscriptome data from IBD patients and healthy volunteers in the IBDMDB were used to retrieve the polyamine biosynthetic genes based on the EC numbers obtained in the BLASTP analysis (Table S5). As shown in Figure 6e, the transcriptome levels of speA, speC, and TTHA0824 were significantly higher in IBD patients compared to healthy subjects. Correlation analysis revealed positive associations between polyamines and enzyme transcription levels. Specifically, putrescine was positively correlated with the transcription levels of speA and speC, while N1-acetylspermidine was positively correlated with the transcription levels of TTHA1129 and arginase (Figure 6e). The findings suggest that elevated transcription levels of polyamine biosynthesis enzymes in the gut microbiome could be associated with the increased levels of polyamines observed in IBD patients.

### Biosynthesis of Polyamines in Bacteroides

**Figure 7.**
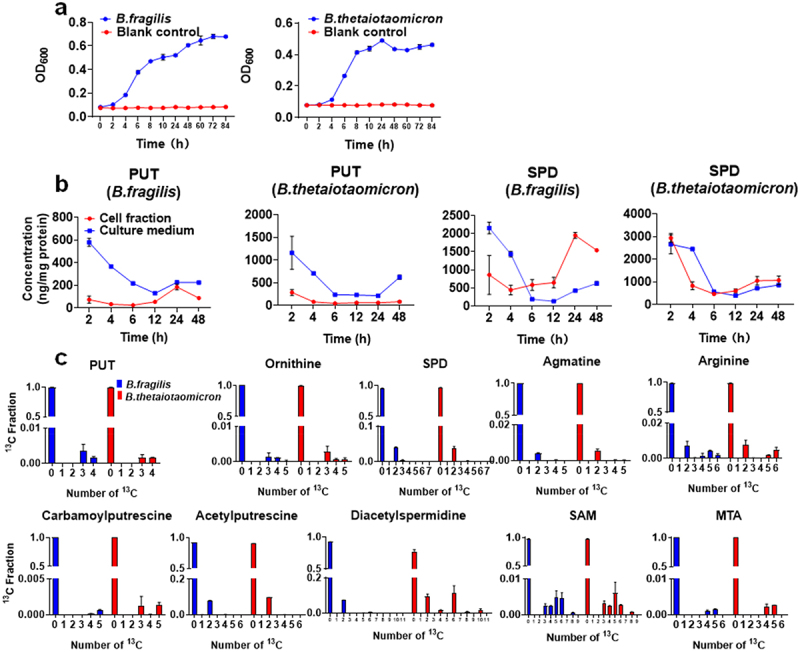
Contribution of *Bacteroides* spp. To polyamine biosynthesis. Panel (a) growth curve of *B. fragilis* and *B. thetaiotaomicron*. Panel (b) time-dependent changes of PUT and SPD levels in the culture medium and cell fraction of *Bacteroides* spp. Samples were analyzed by LC-MS/MS and the data were normalized by protein contents. Panel (c) ^13^C fractional enrichment of polyamines and related metabolites in *Bacteroides* spp. after incubation with [U-^13^C]-inulin for 24 h. Values shown are mean ± SEM (*n* = 3).

On the basis of the IBDMDB, *Bacteroides* was identified as a major genus in the human gut microbiome that harbors the polyamine biosynthesis enzymes. Therefore, *B. fragilis* and *B. thetaiotaomicron*, the major *Bacteroides* species in the human gut, were used as representative strains to investigate the biosynthesis of PUT and SPD. The polyamine levels in *Bacteroides* spp. were firstly determined using LC-MS/MS. These two bacteria were cultured separately for up to 84 h, and the culture medium as well as cell fraction at different time points were analyzed to quantify PUT and SPD. The growth of *B. fragilis* and *B. thetaiotaomicron* were in the lag phase at 2 h, then entered the logarithmic phase at 24 h, and remained in the stationary phase at 48 h (Figure 7a). Accordingly, six sampling time points were designed for the determination of polyamines in the bacteria. In *B. fragilis* and *B. thetaiotaomicron*, the concentration of PUT in the culture medium decreased gradually along with the time. The concentration of PUT in the cell fraction of *B. fragilis* increased gradually, and a peak level was observed at 24 h, which may contribute to the slightly elevated PUT in the culture medium. Interestingly, the level of PUT in the culture medium was consistently higher than that in the cell fraction for both bacteria. The highest level of SPD in the cellular fraction of *B. fragilis* was observed at 24 h in the logarithmic growth phase, which was in concurrence with the increased SPD in the culture medium. For *B. thetaiotaomicron*, the level of SPD was higher in the culture medium than the cells at 4 h, and the reverse was true after 6 h of incubation (Figure 7b). Consistent with previous reports,^50,55^ *Bacteroides* were identified as the major bacteria expressing polyamine transporters (Figure S5), which may contribute to the different levels of polyamines between the culture medium and the cell fraction of the fecal microbiome.

Milovic et al. reported that PUT excretion from Caco-2 cells into the basolateral medium did not exceed 50 pM.^56^ Additionally, Weiss et al. found that the highest concentrations of PUT and SPD in the intestinal epithelial cells of patients with ulcerative colitis (UC) and Crohn’s disease (CD) were less than 1 nmol/mg and 5 nmol/mg, respectively.^9^ In our study, we found that the PUT and SPD in the culture media of *Bacteroides* spp. were 109–520 nM and 112–765 nM (Figure S6), or 130–1162 and 135–2660 ng/mg protein (Figure 7b). Taken together, the amount of polyamine released by intestinal epithelial cells is lower than that released from *Bacteroides* spp, suggesting that gut bacteria are major contributors to fecal polyamine.

SIRM analysis was used to investigate the contribution of *Bacteroides* to polyamines biosynthesis. After a 24-hour incubation of *Bacteroides* spp. with [U-^13^C]-inulin, the resulting ^13^C labeled polyamines were analyzed by Fmoc-derivatization and LC-HRMS. The major isotopologues of PUT in *B. fragilis* and *B. thetaiotaomicron* were ^13^C_3_ and ^13^C_4_, likely originating from ^13^C-labeled ornithine (Figure 7c). In contrast, the predominant isotopologue of SPD was ^13^C_2_, accounting for 3.9% and 3.8% of the total carbon pool in *B. fragilis* and *B. thetaiotaomicron*, respectively. Similarly, ^13^C_2_ was the major isotopologue for agmatine in both strains, representing 0.4% and 0.6% of the total carbon pool, respectively. Additionally, in both bacterial species, the major isotopologue for carbamoylputrescine was ^13^C_5_, and for acetylputrescine and diacetylspermidine, it was ^13^C_2_, aligning with findings from human fecal microbiome incubations (Figure 5). Further analysis of polyamine biosynthesis related metabolites revealed that the major isotopologue of SAM and MTA was ^13^C_5_ in both bacterial species (Figure 7c), consistent with results of human fecal microbiome incubations (Figure S2b). These results further supported that *Bacteroides* spp. contributed to the biosynthesis of PUT and SPD, with the arginine-agmatine-SPD pathway predominating over the ornithine-PUT-SPD pathway in SPD production within *Bacteroides*.

## Phylogenetic analysis of enteric bacterial SPD biosynthesis genes

**Figure 8.**
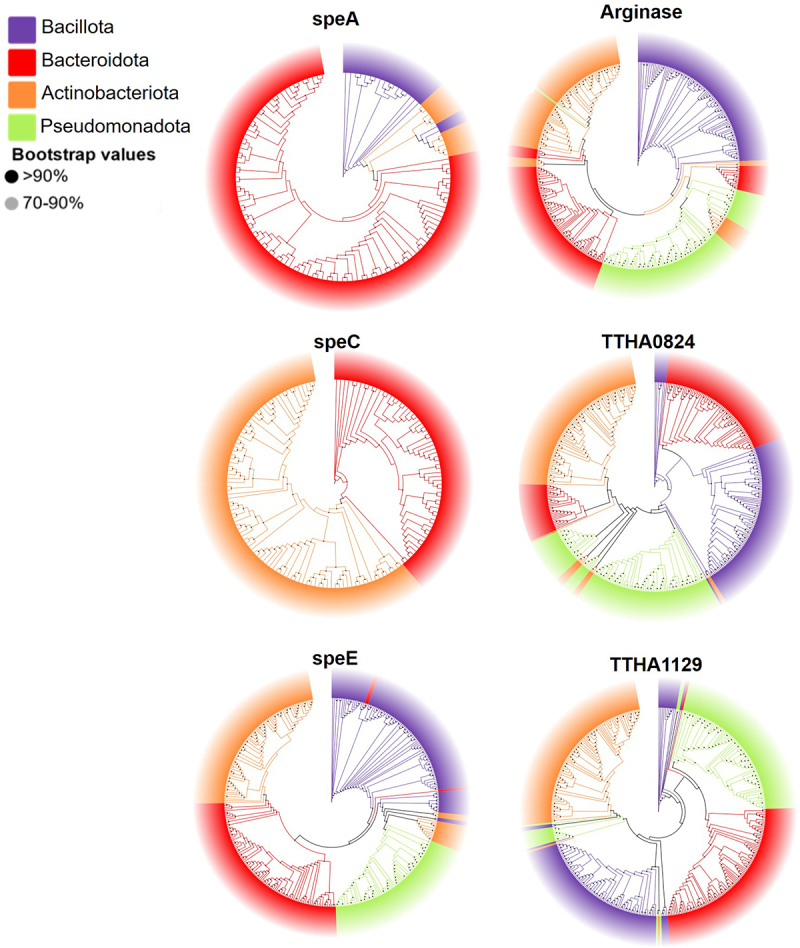
Phylogenetic tree of gut bacteria express genes coding SPD biosynthesis enzymes including arginase, speA, speC, speE, TTHA0824, and TTHA1129, constructed using the NCBI Prokaryotic RefSeq Genomes database. The color coding (purple, red, orange, and green) represents different bacterial phyla, including Bacillota, Bacteroidota, actinobacteriota, and Pseudomonadota.

To evaluate the evolutionary relationships among polyamine-producing intestinal bacteria, a phylogenetic analysis was conducted using the Prokaryotic RefSeq Genomes database (https://www.ncbi.nlm.nih.gov/refseq/about/prokaryotes/). The bacteria selected for phylogenetic analysis included Bacillota, Bacteroidota, Actinobacteriota and Pseudomonadota, which represent the four major bacterial phyla in the human gut microbiome.^57^ This analysis, based on genome read data, allows for a comprehensive comparison of all clades without requiring the merging of taxa at predefined taxonomic levels, thereby offering a more nuanced understanding of microbial community. Enzymes involved in both the ornithine-PUT-SPD and arginine-agmatine-SPD pathways were used as queries (Table S7 and S8). The resulting phylogenetic trees are showed in Figure 8, which represented the microbial diversity of specific gene sequences associated with different gut microbiome bacterial taxa. Bacterial species are depicted as external nodes interconnected by multiple branches through internal nodes. The branching patterns suggested the relationships between these bacteria, and the bootstrap values (indicated by shaded circles at each node) provided statistical support for these relationships. For speA, a tree dominated by red branches indicated a higher abundance of Bacteroidota in the expression of speA genes compared to other bacterial phyla. Further analysis of the genetic distance demonstrated that Bacteroidota have minimal species variation in speA gene (Figure S7). On the contrary, the long branches observed in the Actinobacteriota and Bacillota sections indicated significant genetic divergence among these bacterial groups. The phylogenetic analysis further underscored the significance of Bacteroidota in SPD biosynthesis.

## Discussion

Polyamines are recognized as important metabolites for human intestinal health and disease.^58^ However, the role of the gut microbiome in polyamine biosynthesis of and its implications in IBD remain poorly understood. To address this gap, we developed a derivatization-LC-HRMS method and applied it to investigate polyamine biosynthesis in the gut microbiome. This method, when combined with our previously established HILIC-HRMS based SIRM approaches,^27,28^ provided sufficient sensitivity and comprehensive metabolite coverage, enabling the precise determination of the ^13^C isotopologue profiles of PUT, SPD and related biochemicals. Although a previous study using ^13^C, ^15^N-fully labeled arginine as a stable isotope tracer provided valuable contributions to understanding of biosynthesis of putrescine,^26^ the use of a single metabolite precursor is insufficient to capture the complexity of interconnected metabolism pathways. Therefore, in this study, we employed [U-^13^C]-inulin as a tracer, which is a preferred substrate for the gut microbiome, and therefore allowed for a more holistic tracking of metabolic pathways, leading to the discovery of a novel SPD biosynthetic route.

It has been well acknowledged that aminopropylation of PUT via spermidine synthase is a major pathway for the SPD biosynthesis, where PUT serves as the precursor for SPD.^24,59^ Our study identified arginine-agmatine-SPD as an alternative SPD biosynthesis pathway in human gut microbiome, as demonstrated by human fecal microbiome incubation studies using [U-^13^C]-inulin as the tracer. Both PUT and SPD were found to be *de novo* synthesized in the human gut microbiome (Figures 3b and 5). Notably, a significant difference in the total ^13^C fraction (∑^13^C) was observed, with the ∑^13^C of SPD accounting for 42.8% compared to 2.8% for PUT (Figure 5). In addition, ^13^C_4_ was the predominant isotopologue of PUT, whereas ^13^C_2_ was the major isotopologue of SPD. These findings strongly suggested distinct biosynthetic pathways for SPD and PUT. Arginine-agmatine-SPD was reported as an alternative pathway to produce SPD in thermophilic bacterium and archaea.^51,52^ By tracking ^13^C metabolism through this pathway, we observed a substantially higher fractional ^13^C enrichment in ^13^C_2_-agmatine compared to other isotopologues, suggesting agmatine’s potential role as a precursor in SPD biosynthesis. Further *in silico* analysis and single-strain SIRM study demonstrated that Bacteroidota harbored all the key genes encoding enzymes required for polyamine biosynthesis, including arginase, speA, speC, speE, TTHA0824, and TTHA1129 (Figure 8). Specifically, *B. fragilis* and *B. thetaiotaomicron*, the major *Bacteroides* spp. in human gut, produced ^13^C_2_-labeled SPD as the major isotopologue, similar to that observed in the human fecal microbiome, therefore highlighting the capacity of *Bacteroides* spp. to independently synthesize SPD. Given the higher enrichments of ^13^C_2_-SPD and ^13^C_2_-agmatine compared to other ^13^C isotopologues in both the gut microbiome and *Bacteroides* spp., we propose that the arginine-agmatine-SPD pathway is important in gut microbial SPD biosynthesis, but further large-scale clinical studies are needed to investigate polyamine biosynthetic pathways in the gut microbiome, considering factors such as population diversity, dietary patterns, and age. In addition, the catabolism by gut microbiome could also affect the isotopologues distribution of polyamines besides biosynthesis. In our SIRM analysis, the catabolism metabolites of polyamines, such as acetylputrescine and diacetylspermidine were detected. However, due to limitations in method sensitivity and the instability of intermediate metabolites, catabolic products such as carboxyspermidine, 4-amino-butanal and 4-amino-butanoate were not captured. Therefore, the SIRM analytical method should be further improved to include metabolites in both the biosynthesis and catabolism pathways.

Cellular metabolites are in dynamic homeostasis, and their ^13^C-labeling reflect the balance of biosynthesis, catabolism, and transport. Although similar ^13^C-labeling patterns were observed for polyamines in intra- and extra-cellular pools (Figure 5), the levels of ^13^C-labeled polyamines in the culture media were lower than those in the cellular fraction by comparing their LC-MS peak areas (Table S9 and S10). This could because of minor amounts of polyamines being released outside the microbes and/or active polyamine uptake into microbial intracellular pool. A previous study reported that agmatine has a high efflux rate in *B. fragilis* and *B. thetaiotaomicron* (60–70%) and exceeds 95% in other strains, including *Enterococcus faecalis*, *Bacteroides vulgatus*, *Lactobacillus reuteri*, and *Bifidobacterium bifidum*.^60^ In our SIRM analysis, the fractional enrichment of ^13^C_2_-agmatine in the culture medium of the human fecal microbiome was 4.4%, significantly higher than the 1.8% observed in the cellular fraction (Figure 5b and S3). On the contrary, the fractional enrichment of ^13^C_2_-SPD in the culture medium of the human fecal microbiome was significantly lower than that in the cellular fraction. These findings support the high efflux rate of agmatine and may explain the lower fractional enrichment of ^13^C_2_-agmatine compared to ^13^C_2_-SPD in the intracellular pool. Based on the results of the SIRM study, we cannot rule out the possibility that other precursors contribute to ^13^C_2_-SPD production, and further investigations are needed to clarify the pathway. It should also be noted that the ^13^C_2_ fractional enrichment of SPD in the *Bacteroides* spp. was significantly lower than that in the fecal microbiome (3.9% vs 32.2%). Considering that the genes coding enzymes required for the SPD biosynthesis are widely distributed among gut microbiome phyla (Figure 8), it is plausible that potential hybrid systems exist for the production of SPD in gut microbial communities, similar to what have been found for putrescine biosynthesis.^25,26^ Future studies on the metabolism system orchestrated by multiple bacterial species in the production of SPD are warranted.

Complex shifts in microbiota composition are commonly observed in IBD,^30,61^ and gut microbiome-derived metabolites have been implicated in the development and progression of IBD.^62^ In this study, we found that the levels of polyamines in the feces of IBD patients were significantly higher (Figure 6a), likely associated with the upregulation of microbial genes involved in polyamine biosynthesis in IBD patients (Figure 6e). Specifically, *Bacteroides* spp. were found to express genes coding enzymes responsible for polyamine biosynthesis (Figure 8). Supporting this, a previous study showed that urine concentrations of PUT and SPD were higher in IBD patients compared to control individuals.^63^ Additionally, bacteria such as *B. fragilis* were found to be consistently enriched in six IBD cohorts,^61^ potentially contributing to enhanced polyamine biosynthesis due to its polyamine-producing capacity, as demonstrated in our study (Figure 7). However, it is unclear whether the elevation of polyamine is a contributing factor to the disease or a secondary response to the inflammatory state. This ambiguity underscores the need for further mechanistic studies to delineate causality. In order to further explore the role of polyamine in IBD, studies should employ a method that can separate gut microbial cells from intestinal luminal contents to independently measure intracellular and extracellular polyamine concentrations in human feces. This approach will allow clarification of the contributions of host-derived and microbiome-derived polyamines to IBD, and further help determine the relationship between polyamine and IBD pathophysiology. Moreover, in the current SIRM analysis, we did not detect ^13^C-labeled spermine due to its presence at trace levels and the relatively low detection response after Fmoc-OSu derivatization. However, this does not definitively indicate that the gut microbiome cannot produce spermine. To address this, future studies should focus on developing more sensitive methods capable of capturing trace amounts of spermine, enabling a comprehensive analysis of its isotope labeling.

Although human and murine gut microbiome share 90% and 89% similarities in phyla and genera, respectively, there are key differences in microbial composition and abundance. The most notable distinction is in the ratio of the two major phyla: humans have a relatively high ratio of Bacillota/Bacteroidota, whereas the opposite is true for mice. In humans, Bacteroidota primarily consists of the Bacteroidaceae and Prevotellaceae families, and Bacillota mainly comprises Ruminococcaceae.^64^ Conversely, the mouse gut microbiome is dominated by Bacteroidota, specifically the Muribaculaceae family, which accounts for 54.99–83.44% of the microbial community.^65^ Muribaculaceae are known to produce a wide array of carbohydrate-hydrolyzing enzymes,^66^ which could result in a higher efficiency in metabolizing ^13^C-inulin, and may partially explain the relative higher ^13^C-fractional enrichment of some polyamines and related metabolites in the mouse fecal microbiome (Figure 5). Therefore, species differences need to be considered when translating the polyamine data involving gut microbiomes from rodents to humans. Nevertheless, mouse models remain useful for studying polyamine pathways, as the major ^13^C isotopologues of SPD and PUT found in human fecal microbiome were also observed in mice.

## Conclusions

In summary, we developed a chemical derivatization-based SIRM strategy to investigate polyamine biosynthesis in human gut microbiome. Using [U-^13^C]-inulin as a tracer, we uncovered the *de novo* synthesis of SPD and PUT. Notably, we found that the arginine-agmatine-SPD pathway contributes to the SPD biosynthesis. *In silico* analysis and SIRM studies with bacterial single strains revealed that *Bacteroides* spp. were key contributors to polyamine biosynthesis. By leveraging metabolomic and metatranscriptomic data of an IBD database, polyamines were identified as significantly elevated metabolites in IBD patients, potentially linked to gut dysbiosis and altered bacterial transcript abundances. This study uncovers a novel role of the gut microbiome in polyamine biosynthesis, and further research is warranted to investigate the factors influencing polyamine metabolism and its potential implications in intestinal diseases.

## Supplementary Material

Supplemental Material

## Data Availability

Raw metabolomics data have been deposited in the Metabolomics Workbench and the DOI for this project (PR002151) is: http://dx.doi.org/10.21228/M8KG00. The LC-MS peak areas of the metabolites are listed in Tables S9-S13.
