# Supplementary material for: Uncovering de novo polyamine biosynthesis in the gut microbiome and its alteration in inflammatory bowel disease
Source: Gut Microbes. 2025 Feb 9;17(1):2464225. doi: 10.1080/19490976.2025.2464225 (PMC11812404; doi:10.1080/19490976.2025.2464225)
Supplement: Supplemental Material [file KGMI_A_2464225_SM7047.docx]

**Supplementary Information**

**Uncovering *de novo* polyamine biosynthesis in the gut microbiome and its alteration in inflammatory bowel disease**

Xinwei Li^1,2^, Xia Xiao^1,2^, Shengnan Wang^1,2^, Biyu Wu^1,2^, Yixuan Zhou^1,2^, Pan Deng^1,2^

^1^ Jiangsu Key Laboratory of Neuropsychiatric Diseases and College of Pharmaceutical Sciences, Soochow University, Suzhou, Jiangsu, China

^2^ Department of Pharmaceutical Analysis, Soochow University, Suzhou, Jiangsu, China

Table S1. The demographic information of IBD patients and non-IBD controls included in the current study. Data were collected from the Inflammatory Bowel Disease Multi-omics Database (IBDMDB, <https://ibdmdb.org/>).

| Variable | No-IBD (N = 135) | IBD (N=135) | p. value |  |
| --- | --- | --- | --- | --- |
|  |  |  |  |  |
| Age, median, (25%-75%) yrs | 23 (13-53) | 15 (13-32) | 0.024 ^a^ |  |
| Gender, Male, N (%) | 74 (54.81) | 73 (54.07) | 0.903 ^b^ |  |
| Alcohol, yes, N (%) | 51 (37.78) | 44 (32.59) | 0.372 ^b^ |  |
| Diarrhea, yes, N (%) | 31 (22.96) | 66 (48.89) | <0.001 ^b^ |  |

^a^ Student’s t–test.

^b^ Chi square test.

Table S2. The Clinical characteristics of IBD patients. Data were collected from the Inflammatory Bowel Disease Multi-omics Database (IBDMDB, <https://ibdmdb.org/>).

| Variable | CD (N = 87) | UC (N=48) | p. value |  |
| --- | --- | --- | --- | --- |
|  |  |  |  |  |
| Age, median, (25%-75%) yrs | 15 (13-28) | 16 (13-30) | 0.220 ^a^ |  |
| <10 yrs, N (%) | 9 (10.34) | 4 (8.33) | 0.705 ^b^ |  |
| 10-18 yrs, N (%) | 45 (51.72) | 21 (43.75) | 0.375 ^b^ |  |
| >18 yrs, N (%) | 33 (37.93) | 23 (47.92) | 0.260 ^b^ |  |
| Gender, Male, N (%) | 52 (59.77) | 21 (43.75) | 0.074 ^b^ |  |
| Alcohol, yes, N (%) | 21 (24.14) | 21 (43.75) | 0.018 ^b^ |  |
| Bowel surgery, yes, N (%) | 16 (18.39) | 1 (2.08) | 0.006 ^b^ |  |
| Diarrhea, yes, N (%) | 44 (50.57) | 22 (45.83) | 0.598 ^b^ |  |
| Fecal Cal (μg/g) ^c^, N (%) |  |  |  |  |
| 0-50 | 21 (24.14) | 13 (27.08) | 0.706 ^b^ |  |
| 50-250 | 25 (28.74) | 17 (35.42) | 0.422 ^b^ |  |
| >250 | 17(19.54) | 9 (18.75) | 0.911 ^b^ |  |
| Unknown | 24 (27.59) | 9 (18.75) | 0.253 ^b^ |  |
| SCCAI ^d^, N (%) |  |  |  |  |
| <5 | 3 (3.45) | 43 (89.58) | <0.001 ^b^ |  |
| ≥5 | 1 (1.15) | 4 (8.33) | 0.034 ^b^ |  |
| Unknown | 83 (95.40) | 1 (2.08) | <0.001 ^b^ |  |
| Colonic disease extent ^e^, N (%) |  |  |  |  |
| L1 | 22 (25.29) |  |  |  |
| L2 | 8 (9.20) | 4 (8.33) | 0.866 ^b^ |  |
| L3 | 52 (59.77) |  |  |  |
| L4 | 41 (47.13) | 4 (8.33) | <0.001 ^b^ |  |
| Unknown | 5 (5.75) | 44 (91.67) | <0.001 ^b^ |  |
| HBI ^f^, N (%) |  |  |  |  |
| <5 | 68 (78.16) | 1 (2.08) | <0.001 ^b^ |  |
| ≥5 | 9 (10.34) |  |  |  |
| Unknown | 10 (11.50) | 47 (97.92) | <0.001 ^b^ |  |
| Extraintestinal Manifestations, N (%) |  |  |  |  |
| Arthralgia | 3 (3.45) | 0 (0.00) |  |  |
| Aphthous ulcers | 1 (1.15) | 0 (0.00) |  |  |

^a^ Student’s t–test.

^b^ Chi square test.

^c^ Fecal Cal, Fecal Calprotectin. 0 - 50 μg/g represents the normal level or the remission period, 50 - 250 μg/g represents mild inflammation or the transition stage from the remission period to the active period, >250 μg/g represents the active period of inflammation, and "Unknown" represents the missing data.

^d^ SCCAI, Simple Clinical Colitis Activity Index, values reflect the disease activity level of patients with UC. SCCAI < 5 indicates the remission period, ≥ 5 indicates the active period, and "Unknown" represents the missing data.

^e^ Classified according to Montreal classification. L1, Ileum; L2, Colon; L3, Ileocolonic; L4, Upper GI.

^f^ HBI, Harvey-Bradshaw index. Values reflect the disease activity level of patients with CD. HBI < 5 indicates the remission period, ≥ 5 indicates the active period, and "Unknown" represents the missing data.

Table S3. List of polyamines and amino acids detected by the Fmoc-OSu derivatization combined with the LC-MS analysis.

| Metabolite | Formula | [M+H]^+^ | Retention time (min) |
| --- | --- | --- | --- |
| [Fmoc]_1_-SPD | C22H29O2N3 | 368.23 | 4.52/4.66 |
| [Fmoc]_2_-SPD | C37H39O4N3 | 590.30 | 7.53 |
| [Fmoc]_3_-SPD | C52H49O6N3 | 812.36 | 13.74 |
| [Fmoc]_1_-PUT | C19H22O2N2 | 311.17 | 5.60/11.40 |
| [Fmoc]_2_-PUT | C34H32O4N2 | 533.24 | 11.40 |
| [Fmoc]_1_-ornithine | C20H22O4N2 | 355.16 | 5.74 |
| [Fmoc]_2_-ornithine | C35H32O6N2 | 577.23 | 10.47 |
| [Fmoc]_1_-citrulline | C21H23O5N3 | 398.17 | 6.63 |
| [Fmoc]_2_-citrulline | C36H33O7N3 | 620.23 | 7.41 |
| [Fmoc]_3_-citrulline | C51H43O9N3 | 842.30 | ND |
| [Fmoc]_1_-arginine | C21H24O4N4 | 397.18 | 5.53 |
| [Fmoc]_1_-agmatine | C20H24O2N4 | 353.19 | 5.86 |
| [Fmoc]_2_-agmatine | C35H34O4N4 | 575.26 | 7.77 |
| [Fmoc]_1_-carbamoyl  putrescine | C20H23N3O3 | 354.18 | 7.21 |
| [Fmoc]_1_-glutamate | C20H19NO6 | 370.13 | 8.61 |
| [Fmoc]_1_-diacetylspermidine | C26H33N3O4 | 452.25 | 6.88 |

Table S4. List of metabolites detected by the HILIC-HRMS method.

| Metabolite | Formula | m/z | Retention time (min) |
| --- | --- | --- | --- |
| Aspartate | C4H7NO4 | 134.04 | 10.02 |
| Asparagine | C4H8N2O3 | 133.06 | 9.32 |
| Threonine | C4H9NO3 | 120.06 | 8.20 |
| Alanine | C3H7NO2 | 90.05 | 8.80 |
| Glycine | C2H5NO2 | 76.03 | 9.76 |
| Leucine/Isoleucine | C6H13NO2 | 132.10 | 5.25 |
| Valine | C5H11NO2 | 118.08 | 5.70 |
| Lysine | C6H14N2O2 | 147.11 | 15.06 |
| Acetyl lysine | C8H16N2O3 | 189.12 | 4.07 |
| Saccharopine | C11H20N2O6 | 277.13 | 10.55 |
| Pipecolic acid | C6H11NO2 | 130.08 | 6.68 |
| Guanidinobutyric acid | C5H11N3O2 | 146.09 | 9.20 |
| Aminovaleric acid | C5H11NO2 | 118.08 | 9.54 |
| Glutamine | C5H10N2O3 | 147.07 | 9.17 |
| Glutamate | C5H9NO4 | 148.06 | 9.23 |
| N-succinyl-L-glutamate | C9H13NO7 | 248.07 | 12.40 |
| Arginine | C6H14N4O2 | 175.11 | 15.66 |
| Citrulline | C6H13N3O3 | 176.10 | 9.92 |
| N-carbamoylputrescine | C5H13N3O5 | 132.11 | 14.72 |
| Proline | C5H9NO2 | 116.07 | 7.14 |
| Ornithine | C5H12N2O2 | 133.09 | 13.86 |
| Acetyl ornithine | C7H14N2O3 | 175.10 | 7.57 |
| Diacetylspermidine | C11H23N3O2 | 230.18 | 9.16 |
| Phenylalanine | C9H11NO2 | 166.08 | 4.63 |
| Tyrosine | C9H11NO3 | 182.08 | 7.20 |
| Methionine | C5H11NO2S | 150.05 | 5.54 |
| Histidine | C6H9N3O2 | 156.07 | 8.62 |
| 2-Aminobutyric acid | C4H9NO2 | 104.07 | 6.42 |
| Pantothenic acid | C9H17NO5 | 220.11 | 4.13 |
| Nicotinamide | C6H6N2O | 123.05 | 4.08 |
| Biotin | C10H16N2O3S | 245.09 | 6.53 |
| Trimethyl lysine | C9H20N2O2 | 189.15 | 13.45 |
| Adenine | C5H5N5 | 136.06 | 4.53 |
| Guanine | C5H5N5O | 152.05 | 6.81 |
| Adenosine | C10H13N5O4 | 268.10 | 4.29 |
| Guanosine | C10H13N5O5 | 284.09 | 7.28 |
| Hypoxanthine | C5H4N4O | 137.04 | 5.07 |
| Cytosine | C4H5N3O | 112.05 | 6.54 |
| Uracil | C4H4N2O2 | 113.03 | 4.95 |
| Gamma-aminobutyric acid | C4H9NO2 | 104.07 | 9.44 |
| Betaine | C5H11NO2 | 118.08 | 5.72 |
| Choline | C5H14NO | 104.10 | 12.30 |
| S -Adenosylmethionine | C15H23O5N6S+ | 399.14 | 10.21 |
| Methylthioadenosine | C11H15O3N5S | 298.09 | 4.08 |
| N-Acetylputrescine | C6H14ON2 | 131.12 | 11.90 |
| Dimethylglycine | C4H9NO2 | 104.07 | 6.35 |
| Aminobenzoic acid | C7H7NO2 | 138.05 | 6.14 |
| Glycyl-L-leucine | C8H16N2O3 | 189.12 | 4.07 |
| Acetyl aspartate | C6H9NO5 | 176.05 | 9.97 |
| Ethanolamine | C2H7NO | 62.06 | 12.88 |
| Glucose 6-phosphate | C6H12O9P | 261.03 | 11.93 |
| Lactate | C3H6O3 | 89.02 | 4.07 |
| Propionate | C3H6O2 | 73.02 | 4.02 |
| Butyrate | C4H8O2 | 87.04 | 4.00 |
| Hydroxyglutarate | C5H8O5 | 147.02 | 10.29 |
| Succinate | C4H6O4 | 117.01 | 10.26 |
| Fumarate | C4H4O4 | 115.00 | 11.18 |
| Malate | C4H6O5 | 133.01 | 10.97 |
| Pyruvate | C3H4O3 | 87.00 | 4.00 |
| Phosphoenol pyruvate | C3H5O6P | 166.97 | 12.37 |
| Alpha-ketoglutarate | C5H6O5 | 145.01 | 10.53 |
| 3-Phosphoglyceric acid | C3H7O7P | 18.98 | 12.02 |
| Glycerol-3-phosphate | C3H9O6P | 171.00 | 9.99 |

Table S5. List of proteins with sequences closely related to polyamine metabolism enzymes identified in the RefSeq Select proteins Database.

| Protein name | Highly similar protein name | GenBank accession no. | EC no. |
| --- | --- | --- | --- |
| TTHA1129 | agmatinase | WP_023295409 | 3.5.3.11 |
|  | formimidoylglutamase | WP_047732962 | 3.5.3.8 |
|  | arginase | NP_000036 | 3.5.3.1 |
| TTHA0824 | polyamine aminopropyltransferase | WP_013319061 | 2.5.1.16 |
|  | spermine synthase | NP_001245352 | 2.5.1.22 |
|  | 16S rRNA (cytosine(967)-C(5))-methyltransferase | WP_229222743 | 2.1.1.176 |
|  | 23S rRNA (cytosine(1962)-C(5))-methyltransferase | WP_023330120 | 2.1.1.191 |
|  | peptide chain release factor N(5)-glutamine methyltransferase | WP_142518715 | 2.1.1.297 |
|  | tRNA1(Val) (adenine(37)-N6)-methyltransferase | WP_086524710 | 2.1.1.223 |
|  | methyltransferase-like protein | NP_001025162 | 2.1.1.72 |
|  | guanidino acid hydrolase | NP_079034 | 3.5.3.7 |
| speA | arginine decarboxylase | NP_195197 | 4.1.1.19 |
|  | ornithine decarboxylase | NP_001274117 | 4.1.1.17 |
|  | diaminopimelate decarboxylase | WP_227757131 | 4.1.1.20 |
| Arginase | arginase | NP_000036 | 3.5.3.1 |
|  | agmatinase | WP_154948163 | 3.5.3.11 |
|  | guanidino acid hydrolase | NP_079034 | 3.5.3.7 |
| speC | ornithine decarboxylase | NP_002530 | 4.1.1.17 |
|  | PLP-dependent decarboxylase | WP_193988155 | 4.1.1.81 |
|  | alanine racemase | WP_106660928 | 5.1.1.1 |
|  | diaminopimelate decarboxylase | WP_226571462 | 4.1.1.20 |
|  | hypothetical protein | WP_131002677 | 6.3.4.23 |
| speE | spermidine synthase | NP_003123 | 2.5.1.16 |

Table S6. List of transporter proteins involved in polyamine uptake or export.

| Gene ID | GenBank  accession no. | Gene ID | GenBank  accession no. |
| --- | --- | --- | --- |
| plaP | NP_416518 | potD | NP_415641 |
| puuP | NP_415812 | aguD | NP_814482 |
| potF | NP_415375 | sapB | NP_415809 |
| potG | NP_415376 | sapC | NP_415808 |
| potH | NP_415377 | sapD | NP_415807 |
| potI | NP_415378 | sapF | NP_415806 |
| potA | NP_415644 | potE | NP_415219 |
| potB | NP_415643 | mdtJ | NP_416117 |
| potC | NP_415642 | mdtI | NP_416116 |

Table S7. List of enzymes involved in the ornithine-PUT-SPD pathway.

| Protein name | Description | GenBank accession no. |
| --- | --- | --- |
| Arginase | arginase | NP_000036 |
| speC | ornithine decarboxylase | BAI37506 |
| speE | spermidine synthase | AEE54785 |

Table S8. List of enzymes involved in the arginine-agmatine-SPD pathway.

| Protein name | Description | GenBank accession no. |
| --- | --- | --- |
| speA | arginine decarboxylase | BAI37476 |
| TTHA0824 | agmatine aminopropyltransferase | SDF28732 |
| TTHA1129 | N1-aminopropylagmatine ureohydrolase | Q5SJ85 |

Table S9. LC-HRMS peak areas of polyamines and related metabolites in the cellular fraction of the fecal microbiome incubated with [U-^13^C]-inulin.

| Metabolite | Formula | Isotopologue | mouse 1 | mouse 2 | mouse 3 | human 1 | human 2 | human 3 |
| --- | --- | --- | --- | --- | --- | --- | --- | --- |
| [Fmoc]2-PUT | C34H32O4N2 | M0 | 8403072 | 3462378 | 475682 | 148802560 | 75181507 | 45656318 |
|  |  | M1 | 3168926 | 1032827 | 25324 | 54152708 | 27564904 | 16910057 |
|  |  | M2 | 326936 | 28992 | 0 | 11080165 | 5143321 | 3194343 |
|  |  | M3 | 19410 | 0 | 0 | 1839370 | 877875 | 329548 |
|  |  | M4 | 105477 | 48972 | 0 | 4450791 | 2484420 | 1101847 |
| [Fmoc]3-SPD | C52H49O6N3 | M0 | 3652960 | 4155317 | 3097299 | 34133264 | 6970947 | 3010263 |
|  |  | M1 | 797143 | 1601318 | 746197 | 20930521 | 4253611 | 1137915 |
|  |  | M2 | 3425253 | 2873782 | 2577055 | 26369881 | 5428444 | 1880440 |
|  |  | M3 | 2310303 | 1910569 | 1774206 | 14074551 | 2773418 | 892412 |
|  |  | M4 | 485138 | 553410 | 534976 | 5722429 | 688335 | 32721 |
|  |  | M5 | 1250566 | 1544451 | 1614199 | 8291945 | 791611 | 143357 |
|  |  | M6 | 655504 | 967187 | 830360 | 5594841 | 612513 | 75657 |
|  |  | M7 | 28042 | 52745 | 22466 | 2423663 | 116280 | 10958 |
| [Fmoc]1-arginine | C21H24O4N4 | M0 | 560403418 | 660904560 | 638030703 | 99533765 | 63546589 | 30068091 |
|  |  | M1 | 6251797 | 9729646 | 63038676 | 11172633 | 3426670 | 5571134 |
|  |  | M2 | 22916795 | 24007167 | 23647232 | 3413237 | 2272523 | 1037044 |
|  |  | M3 | 11184978 | 9262719 | 10450129 | 575741 | 378010 | 139130 |
|  |  | M4 | 3724451 | 3518740 | 4218998 | 1036204 | 741570 | 190119 |
|  |  | M5 | 3014706 | 1801931 | 2324137 | 932998 | 719154 | 182089 |
|  |  | M6 | 1826499 | 1579536 | 1889587 | 438401 | 272468 | 58019 |
| [Fmoc]1-agmatine | C20H24O2N4 | M0 | 3634755 | 11170411 | 4759714 | 65557775 | 7943108 | 4881601 |
|  |  | M1 | 77698 | 1218630 | 502549 | 6215924 | 386911 | 876164 |
|  |  | M2 | 81848 | 354566 | 109393 | 1943777 | 104580 | 25664 |
|  |  | M3 | 90800 | 565696 | 265547 | 67852 | 19507 | 4805 |
|  |  | M4 | 0 | 42027 | 50375 | 145186 | 18113 | 5802 |
|  |  | M5 | 11794 | 21174 | 12723 | 511751 | 5386 | 5154 |
| [Fmoc]1-carbamoylputrescine | C20H23N3O3 | M0 | 5885608 | 2704767 | 1565872 | 5558211 | 2820842 | 3005035 |
|  |  | M1 | 901359 | 328297 | 184359 | 1017089 | 356132 | 374968 |
|  |  | M2 | 18793 | 0 | 0 | 0 | 0 | 0 |
|  |  | M3 | 0 | 0 | 0 | 0 | 0 | 0 |
|  |  | M4 | 24343 | 0 | 0 | 15107 | 0 | 0 |
|  |  | M5 | 0 | 0 | 0 | 91404 | 78641 | 111404 |
| [Fmoc]1-citrulline | C21H23O5N3 | M0 | 87708744 | 112406547 | 73016173 | 156309607 | 78764612 | 82870737 |
|  |  | M1 | 20900971 | 26887621 | 18124810 | 35894061 | 18039262 | 19098048 |
|  |  | M2 | 3360125 | 4701441 | 3292686 | 5728313 | 2761351 | 2954014 |
|  |  | M3 | 0 | 0 | 0 | 67558 | 105700 | 93978 |
|  |  | M4 | 227966 | 915095 | 897029 | 1452896 | 761672 | 513366 |
|  |  | M5 | 555617 | 878278 | 783572 | 1158489 | 621229 | 493742 |
|  |  | M6 | 19273 | 157748 | 236477 | 150961 | 99175 | 13469 |
| [Fmoc]1-ornithine | C20H22O4N2 | M0 | 75783812 | 97541291 | 64475620 | 418931986 | 107803472 | 63682002 |
|  |  | M1 | 16202234 | 20592161 | 13518466 | 89408834 | 22670427 | 13114817 |
|  |  | M2 | 2570830 | 3380646 | 2549955 | 11231759 | 2760435 | 1529813 |
|  |  | M3 | 1184374 | 1506172 | 1375277 | 1358961 | 318020 | 115892 |
|  |  | M4 | 498864 | 839947 | 1023049 | 2218074 | 665176 | 198065 |
|  |  | M5 | 1832347 | 2352320 | 2151785 | 13721006 | 4268223 | 1706884 |
| [Fmoc]1-acetylputrescine | C21H24N2O3 | M0 | 23682248 | 28302937 | 21825864 | 1857651 | 955296 | 730275 |
|  |  | M1 | 7835016 | 8800373 | 7435608 | 23732 | 24721 | 104883 |
|  |  | M2 | 40319523 | 37193421 | 33473539 | 320983 | 106825 | 89551 |
|  |  | M3 | 6628842 | 7008014 | 5911817 | 0 | 0 | 0 |
|  |  | M4 | 1183913 | 1264930 | 1181480 | 0 | 0 | 0 |
|  |  | M5 | 0 | 0 | 0 | 0 | 0 | 0 |
|  |  | M6 | 900669 | 1015765 | 1255981 | 10427 | 0 | 0 |
| [Fmoc]1-diacetylspermidine | C26H33N3O4 | M0 | 2399386 | 3904564 | 2575233 | 4980051 | 3737277 | 6038974 |
|  |  | M1 | 415493 | 1184830 | 483409 | 1980208 | 1444536 | 2257594 |
|  |  | M2 | 1532551 | 1803394 | 1454802 | 9531583 | 6333324 | 9591641 |
|  |  | M3 | 291710 | 613429 | 524751 | 3775366 | 2338731 | 3644781 |
|  |  | M4 | 941363 | 1202496 | 1100606 | 8228310 | 3814616 | 6607790 |
|  |  | M5 | 119082 | 175754 | 205033 | 2553451 | 961859 | 2134747 |
|  |  | M6 | 101963 | 144896 | 175876 | 3519708 | 952064 | 2510004 |
|  |  | M7 | 0 | 12847 | 11650 | 1261495 | 101630 | 808416 |
|  |  | M8 | 0 | 0 | 10775 | 1347981 | 50183 | 811107 |
|  |  | M9 | 0 | 0 | 12867 | 640583 | 0 | 171957 |
|  |  | M10 | 0 | 0 | 0 | 751629 | 8251 | 197227 |
|  |  | M11 | 0 | 0 | 0 | 28872 | 0 | 13452 |

Table S10. LC-HRMS peak areas of polyamines and related metabolites in the culture media of the fecal microbiome incubated with [U-^13^C]-inulin.

| Metabolite | Formula | Isotopologue | mouse 1 | mouse 2 | mouse 3 | human 1 | human 2 | human 3 |
| --- | --- | --- | --- | --- | --- | --- | --- | --- |
| [Fmoc]1-PUT | C19H22O2N2 | M0 | 5342393 | 634608 | 201276 | 1223758 | 713805 | 803537 |
|  |  | M1 | 992290 | 14458 | 5358 | 180077 | 73038 | 63715 |
|  |  | M2 | 24391 | 0 | 0 | 0 | 0 | 0 |
|  |  | M3 | 0 | 0 | 0 | 0 | 0 | 0 |
|  |  | M4 | 160872 | 15091 | 24261 | 35239 | 106265 | 50014 |
| [Fmoc]1-SPD | C22H29O2N3 | M0 | 583253 | 8322276 | 9902913 | 2526559 | 1694208 | 1508748 |
|  |  | M1 | 9636 | 0 | 0 | 22878 | 10188 | 10377 |
|  |  | M2 | 130099 | 37724 | 93016 | 357569 | 153496 | 198831 |
|  |  | M3 | 0 | 208471 | 341927 | 25114 | 0 | 6337 |
|  |  | M4 | 0 | 0 | 15105 | 7268 | 0 | 52398 |
|  |  | M5 | 26384 | 42886 | 35732 | 0 | 23778 | 61846 |
|  |  | M6 | 15015 | 7790 | 11231 | 10021 | 16992 | 26862 |
|  |  | M7 | 0 | 6401 | 5932 | 9192 | 17596 | 11426 |
| [Fmoc]1-ornithine | C20H22N2O4 | M0 | 4101270 | 3898215 | 3684307 | 3259499 | 3839017 | 3517522 |
|  |  | M1 | 716009 | 670987 | 782930 | 449868 | 658787 | 585383 |
|  |  | M2 | 3809 | 4321 | 0 | 0 | 0 | 5160 |
|  |  | M3 | 0 | 6211 | 9159 | 9139 | 6625 | 0 |
|  |  | M4 | 0 | 0 | 0 | 0 | 0 | 0 |
|  |  | M5 | 42262 | 38897 | 32955 | 23640 | 61250 | 62466 |
| [Fmoc]1-citrulline | C21H23N3O5 | M0 | 661932 | 602119 | 830446 | 2820479 | 2546294 | 2054110 |
|  |  | M1 | 130744 | 27960 | 94459 | 477247 | 383138 | 305342 |
|  |  | M2 | 204985 | 39502 | 108448 | 0 | 12792 | 0 |
|  |  | M3 | 0 | 0 | 17566 | 0 | 0 | 0 |
|  |  | M4 | 114549 | 74720 | 126837 | 12899 | 7210 | 39824 |
|  |  | M5 | 0 | 105636 | 416625 | 13673 | 5158 | 5459 |
|  |  | M6 | 0 | 0 | 0 | 0 | 6124 | 0 |
| [Fmoc]1-agmatine | C20H24N4O2 | M0 | 8171085 | 8781469 | 3233660 | 15306845 | 16713059 | 15561083 |
|  |  | M1 | 0 | 0 | 0 | 0 | 0 | 0 |
|  |  | M2 | 1033883 | 1001710 | 164833 | 529679 | 321240 | 300673 |
|  |  | M3 | 0 | 0 | 0 | 0 | 17864 | 7378 |
|  |  | M4 | 242792 | 66684 | 110792 | 154997 | 346806 | 220514 |
|  |  | M5 | 0 | 12114 | 10055 | 0 | 0 | 8143 |
| [Fmoc]1-diacetylspermidine | C26H33N3O4 | M0 | 5825313 | 723531 | 2038480 | 494679 | 172876 | 396443 |
|  |  | M1 | 1849134 | 158007 | 756749 | 133184 | 30557 | 108883 |
|  |  | M2 | 6892903 | 582172 | 2746659 | 928773 | 792909 | 1037138 |
|  |  | M3 | 1987194 | 164866 | 1034039 | 437011 | 232600 | 350299 |
|  |  | M4 | 4389812 | 794651 | 2780086 | 984062 | 764114 | 946139 |
|  |  | M5 | 1367088 | 18060 | 632459 | 84511 | 85111 | 84613 |
|  |  | M6 | 2210013 | 61811 | 602015 | 137475 | 179864 | 59736 |
|  |  | M7 | 781137 | 0 | 110142 | 8375 | 0 | 0 |
|  |  | M8 | 23536 | 0 | 17079 | 0 | 7520 | 0 |
|  |  | M9 | 0 | 0 | 10391 | 0 | 0 | 4746 |
|  |  | M10 | 0 | 0 | 0 | 0 | 7010 | 0 |
|  |  | M11 | 0 | 0 | 0 | 0 | 0 | 0 |
| [Fmoc]1-acetylputrescine | C21H24N2O3 | M0 | 63674897 | 1.27E+08 | 1.6E+08 | 1886622 | 1287310 | 1850959 |
|  |  | M1 | 25629300 | 43017136 | 57578564 | 499781 | 258177 | 475363 |
|  |  | M2 | 1.66E+08 | 1.82E+08 | 2.67E+08 | 5619172 | 2976514 | 5776811 |
|  |  | M3 | 32611306 | 36972030 | 53812063 | 985629 | 468393 | 913843 |
|  |  | M4 | 6505237 | 10164081 | 15750020 | 21981 | 7941 | 8205 |
|  |  | M5 | 1518903 | 2789871 | 5498322 | 0 | 0 | 6360 |
|  |  | M6 | 8383516 | 8478732 | 15882393 | 0 | 8854 | 6610 |

Table S11. HILIC-HRMS-ESI(+) peak areas of metabolites in the cellular fraction of the human fecal microbiome incubated with [U-^13^C]-inulin.

| Metabolite | Formula | Isotopologue | Polar_pos1 | Polar_pos2 | Polar_pos3 |
| --- | --- | --- | --- | --- | --- |
| Lysine | C6H14N2O2 | M0 | 1170270775 | 1599527958 | 592979162 |
|  |  | M1 | 78802354 | 108378928 | 39223067 |
|  |  | M2 | 13520576 | 30788345 | 8102808 |
|  |  | M3 | 3968529 | 9632269 | 2142776 |
|  |  | M4 | 13666280 | 32760554 | 8133399 |
|  |  | M5 | 9899442 | 31570289 | 6409290 |
|  |  | M6 | 1471758 | 2677276 | 611186 |
| Acetyl-lysine | C8H16N2O3 | M0 | 92677095 | 121844105 | 35225897 |
|  |  | M1 | 10132616 | 12833009 | 3600094 |
|  |  | M2 | 3516713 | 3895916 | 1284515 |
|  |  | M3 | 4444651 | 2237813 | 675853 |
|  |  | M4 | 1248675 | 1994672 | 719089 |
|  |  | M5 | 279354 | 644808 | 0 |
|  |  | M6 | 1741069 | 3086066 | 890751 |
|  |  | M7 | 891072 | 1089417 | 145518 |
|  |  | M8 | 17074 | 53462 | 3310 |
| Saccharopine | C11H20N2O6 | M0 | 52407846 | 60765019 | 13662188 |
|  |  | M1 | 6233415 | 7386039 | 1132106 |
|  |  | M2 | 2802295 | 3686074 | 38261 |
|  |  | M3 | 57604 | 125590 | 17530 |
|  |  | M4 | 1177128 | 3176762 | 334624 |
|  |  | M5 | 215019 | 272481 | 6392 |
|  |  | M6 | 18906 | 47714 | 3259 |
|  |  | M7 | 0 | 5239 | 4360 |
|  |  | M8 | 6503 | 8123 | 8475 |
|  |  | M9 | 0 | 3677 | 3196 |
|  |  | M10 | 445836 | 725563 | 12953 |
|  |  | M11 | 5013 | 14436 | 3755 |
| Pipecolic acid | C6H11NO2 | M0 | 149447543 | 233006589 | 159743176 |
|  |  | M1 | 9800305 | 15242719 | 10314399 |
|  |  | M2 | 549701 | 2825101 | 1912906 |
|  |  | M3 | 0 | 64889 | 121147 |
|  |  | M4 | 189672 | 1737393 | 1051916 |
|  |  | M5 | 0 | 0 | 0 |
|  |  | M6 | 2306737 | 8946135 | 2683648 |
| Guanidinobutyric acid | C5H11N3O2 | M0 | 82980555 | 87291964 | 31259577 |
|  |  | M1 | 3107436 | 3140461 | 958648 |
|  |  | M2 | 0 | 109937 | 0 |
|  |  | M3 | 0 | 52008 | 6609 |
|  |  | M4 | 55373 | 12811 | 0 |
|  |  | M5 | 5934783 | 1138267 | 213092 |
| Aminovaleric acid | C5H11NO2 | M0 | 4837413293 | 3957565672 | 1662913069 |
|  |  | M1 | 278530971 | 233360087 | 100633295 |
|  |  | M2 | 25944460 | 21449514 | 8833284 |
|  |  | M3 | 78806 | 0 | 25889 |
|  |  | M4 | 0 | 0 | 0 |
|  |  | M5 | 20572838 | 650730 | 1096835 |
| Glutamine | C5H10N2O3 | M0 | 880384886 | 875994786 | 647355296 |
|  |  | M1 | 60244425 | 55621717 | 41033371 |
|  |  | M2 | 25191142 | 29397619 | 15432995 |
|  |  | M3 | 5969067 | 5401852 | 2647111 |
|  |  | M4 | 6813412 | 12639538 | 6180198 |
|  |  | M5 | 1852507 | 1453183 | 1812442 |
| N-Succinyl-L-glutamate | C9H13NO7 | M0 | 1926319 | 254140 | 2069540 |
|  |  | M1 | 48446 | 0 | 11752 |
|  |  | M2 | 865616 | 359275 | 350371 |
|  |  | M3 | 0 | 0 | 0 |
|  |  | M4 | 38738 | 0 | 29787 |
|  |  | M5 | 3244 | 2869 | 2949 |
|  |  | M6 | 0 | 5227 | 7633 |
|  |  | M7 | 241135 | 30990 | 7936 |
|  |  | M8 | 2204441 | 636137 | 523263 |
|  |  | M9 | 3649 | 3287 | 2798 |
| N-Carbamoylputrescine | C5H13N3O | M0 | 55085995 | 40693947 | 15017018 |
|  |  | M1 | 3789414 | 2651086 | 864112 |
|  |  | M2 | 916004 | 0 | 10270 |
|  |  | M3 | 390588 | 8973 | 5964 |
|  |  | M4 | 1353673 | 232469 | 77318 |
|  |  | M5 | 0 | 0 | 0 |
| Diacetylspermidine | C11H23N3O2 | M0 | 13337383 | 17941970 | 5895379 |
|  |  | M1 | 2148601 | 4560001 | 1035399 |
|  |  | M2 | 14407158 | 30810566 | 9299293 |
|  |  | M3 | 2702359 | 6061307 | 1566243 |
|  |  | M4 | 10524688 | 19446934 | 6325232 |
|  |  | M5 | 2061174 | 2213600 | 771191 |
|  |  | M6 | 3392291 | 2939873 | 1351168 |
|  |  | M7 | 577313 | 535587 | 61072 |
|  |  | M8 | 1033529 | 131886 | 110113 |
|  |  | M9 | 42284 | 11335 | 8253 |
|  |  | M10 | 72776 | 7503 | 5679 |
|  |  | M11 | 408410 | 11097 | 548110 |
| Phenylalanine | C9H11NO2 | M0 | 678194583 | 508854790 | 477530430 |
|  |  | M1 | 68626182 | 52920559 | 50345352 |
|  |  | M2 | 3815708 | 3692607 | 3106593 |
|  |  | M3 | 601499 | 710636 | 515630 |
|  |  | M4 | 2913351 | 2998100 | 1373645 |
|  |  | M5 | 0 | 0 | 0 |
|  |  | M6 | 47308 | 22269 | 3387 |
|  |  | M7 | 267896 | 1662 | 3924 |
|  |  | M8 | 1944955 | 287241 | 311777 |
|  |  | M9 | 1081003 | 16381 | 0 |
| Histidine | C6H9N3O2 | M0 | 1128559441 | 1460633561 | 860054520 |
|  |  | M1 | 62450831 | 76975408 | 46719733 |
|  |  | M2 | 4974998 | 6603498 | 3595268 |
|  |  | M3 | 5725 | 19572 | 6225 |
|  |  | M4 | 0 | 0 | 0 |
|  |  | M5 | 0 | 0 | 0 |
|  |  | M6 | 1357157 | 4740278 | 1091390 |
| Pantothenic acid | C9H17NO5 | M0 | 2851843 | 1839637 | 1491451 |
|  |  | M1 | 228182 | 93008 | 113379 |
|  |  | M2 | 12260 | 20667 | 6906 |
|  |  | M3 | 19967 | 14061 | 0 |
|  |  | M4 | 340321 | 251648 | 63044 |
|  |  | M5 | 6687 | 18073 | 0 |
|  |  | M6 | 0 | 0 | 0 |
|  |  | M7 | 358722 | 593241 | 296649 |
|  |  | M8 | 0 | 4092 | 0 |
|  |  | M9 | 21242 | 42428 | 0 |
| 2-Aminobutyric acid | C4H9NO2 | M0 | 72777061 | 78952271 | 13666593 |
|  |  | M1 | 4746897 | 6709749 | 1023787 |
|  |  | M2 | 17841627 | 40963532 | 5780939 |
|  |  | M3 | 772570 | 1741209 | 171895 |
|  |  | M4 | 8060 | 5445 | 3024 |
| NAD+ | C21H27N7O14P2 | M0 | 14989755 | 17586417 | 5292491 |
|  |  | M1 | 2372870 | 3866711 | 1297427 |
|  |  | M2 | 1936808 | 3210141 | 641017 |
|  |  | M3 | 1461381 | 2237092 | 403541 |
|  |  | M4 | 1637680 | 2726239 | 436973 |
|  |  | M5 | 4266161 | 5105271 | 970764 |
|  |  | M6 | 1988946 | 2719270 | 375656 |
|  |  | M7 | 1869194 | 2080291 | 235991 |
|  |  | M8 | 1707715 | 1473105 | 168319 |
|  |  | M9 | 2806854 | 1961739 | 227275 |
|  |  | M10 | 6945009 | 4056798 | 928564 |
|  |  | M11 | 2047264 | 2224320 | 421336 |
|  |  | M12 | 1609729 | 2720274 | 712480 |
|  |  | M13 | 112341 | 196506 | 6847 |
|  |  | M14 | 1366039 | 1465033 | 187362 |
|  |  | M15 | 1413945 | 2656933 | 325181 |
|  |  | M16 | 66505 | 40469 | 80443 |
|  |  | M17 | 1299748 | 3234290 | 525011 |
|  |  | M18 | 1659189 | 3526648 | 1062785 |
|  |  | M19 | 653242 | 1328550 | 542883 |
|  |  | M20 | 11288 | 22276 | 6750 |
|  |  | M21 | 0 | 8098 | 0 |
| Biotin | C10H16N2O3S | M0 | 5600369 | 4581212 | 3655143 |
|  |  | M1 | 1251774 | 513746 | 380668 |
|  |  | M2 | 769142 | 67376 | 36390 |
|  |  | M3 | 2131310 | 556182 | 291039 |
|  |  | M4 | 10635255 | 3256766 | 2689824 |
|  |  | M5 | 1062360 | 385925 | 150208 |
|  |  | M6 | 3547969 | 1968948 | 728208 |
|  |  | M7 | 207585 | 47552 | 4014 |
|  |  | M8 | 920931 | 868201 | 429678 |
|  |  | M9 | 0 | 3770 | 4095 |
|  |  | M10 | 4275 | 0 | 4602 |
| Trimethyl-lysine | C9H20N2O2 | M0 | 55880455 | 60248825 | 18703879 |
|  |  | M1 | 5140801 | 5936631 | 1672199 |
|  |  | M2 | 132339 | 317285 | 44897 |
|  |  | M3 | 2400 | 2427 | 0 |
|  |  | M4 | 69297 | 202515 | 1667 |
|  |  | M5 | 66872 | 188511 | 3747 |
|  |  | M6 | 0 | 4388 | 0 |
|  |  | M7 | 0 | 2181 | 0 |
|  |  | M8 | 5049 | 3767 | 8476 |
|  |  | M9 | 0 | 10012 | 1937 |
| Adenine | C5H5N5 | M0 | 490380865 | 345691948 | 410191546 |
|  |  | M1 | 38070689 | 39076205 | 32935520 |
|  |  | M2 | 13997842 | 31360362 | 19364676 |
|  |  | M3 | 8395175 | 13672502 | 8179536 |
|  |  | M4 | 5212760 | 8178491 | 6858942 |
|  |  | M5 | 2532603 | 1477716 | 1077283 |
| Guanine | C5H5N5O | M0 | 89622878 | 112289925 | 80309838 |
|  |  | M1 | 4627652 | 6853273 | 5170000 |
|  |  | M2 | 54946 | 246615 | 122434 |
|  |  | M3 | 30208 | 110608 | 150344 |
|  |  | M4 | 6815 | 10180 | 7706 |
|  |  | M5 | 9735 | 13449 | 6554 |
| Adenosine | C10H13N5O4 | M0 | 362879730 | 187959785 | 146345800 |
|  |  | M1 | 57009155 | 30666524 | 22359496 |
|  |  | M2 | 32362293 | 12990204 | 8191824 |
|  |  | M3 | 29456628 | 9185189 | 5985949 |
|  |  | M4 | 46207363 | 13207146 | 9142305 |
|  |  | M5 | 206982445 | 48248797 | 37439847 |
|  |  | M6 | 30902820 | 24750431 | 13222606 |
|  |  | M7 | 22019893 | 35552365 | 22365485 |
|  |  | M8 | 22748853 | 24735556 | 14631217 |
|  |  | M9 | 11441440 | 9731334 | 7577891 |
|  |  | M10 | 6100263 | 1440110 | 1340150 |
| Hypoxanthine | C5H4N4O | M0 | 273001746 | 327634224 | 235249663 |
|  |  | M1 | 19328460 | 24072975 | 16902639 |
|  |  | M2 | 10918368 | 9933514 | 5652877 |
|  |  | M3 | 1735275 | 1536661 | 1401541 |
|  |  | M4 | 216104 | 712569 | 662776 |
|  |  | M5 | 79626 | 107380 | 50232 |
| Cytosine | C4H5N3O | M0 | 25469045 | 14125437 | 9244080 |
|  |  | M1 | 1560584 | 727325 | 1006770 |
|  |  | M2 | 2603557 | 1751700 | 742258 |
|  |  | M3 | 136288 | 60535 | 17204 |
|  |  | M4 | 0 | 0 | 0 |
| Uracil | C4H4N2O2 | M0 | 2037547 | 1768710 | 1409058 |
|  |  | M1 | 6275 | 5344 | 2677 |
|  |  | M2 | 8722 | 0 | 0 |
|  |  | M3 | 0 | 0 | 0 |
|  |  | M4 | 340190 | 708034 | 233263 |
| Betaine | C5H11NO2 | M0 | 4856895735 | 3929727402 | 1731223813 |
|  |  | M1 | 285202277 | 237171141 | 101301450 |
|  |  | M2 | 26062275 | 22275582 | 8584745 |
|  |  | M3 | 394738 | 395485 | 114190 |
|  |  | M4 | 0 | 0 | 0 |
|  |  | M5 | 20572838 | 591063 | 1096835 |
| Choline | C5H14NO | M0 | 173584386 | 249754226 | 80818539 |
|  |  | M1 | 9137933 | 13433672 | 4080319 |
|  |  | M2 | 201381 | 500529 | 8983 |
|  |  | M3 | 0 | 0 | 0 |
|  |  | M4 | 547132 | 166378 | 57058 |
|  |  | M5 | 7155180 | 3523294 | 1776802 |
| Dimethylglycine | C4H9NO2 | M0 | 36009273 | 132813648 | 50872923 |
|  |  | M1 | 2188641 | 5456639 | 2993328 |
|  |  | M2 | 212884 | 510201 | 154966 |
|  |  | M3 | 696343 | 412267 | 802514 |
|  |  | M4 | 7108168 | 3026747 | 3618683 |
| Aminobenzoic acid | C7H7NO2 | M0 | 16979577 | 23807435 | 7782926 |
|  |  | M1 | 1304736 | 1583290 | 845285 |
|  |  | M2 | 0 | 0 | 0 |
|  |  | M3 | 0 | 0 | 0 |
|  |  | M4 | 0 | 0 | 0 |
|  |  | M5 | 0 | 0 | 0 |
|  |  | M6 | 668933 | 1945057 | 152513 |
|  |  | M7 | 7399749 | 16139939 | 3174338 |
| Glycyl-L-leucine | C8H16N2O3 | M0 | 92677095 | 121844105 | 35225897 |
|  |  | M1 | 10132616 | 12833009 | 3600094 |
|  |  | M2 | 3516713 | 3895916 | 1284515 |
|  |  | M3 | 4444651 | 2237813 | 675853 |
|  |  | M4 | 1248675 | 1994672 | 719089 |
|  |  | M5 | 279354 | 644808 | 0 |
|  |  | M6 | 1741069 | 3086066 | 890751 |
|  |  | M7 | 891072 | 1089417 | 188070 |
|  |  | M8 | 17074 | 53462 | 3310 |
| Ethanolamine | C2H7NO | M0 | 1686724 | 1043312 | 623585 |
|  |  | M1 | 10982 | 2057 | 1626 |
|  |  | M2 | 369281 | 117019 | 49560 |
| Glycine | C2H5NO2 | M0 | 50277930 | 55693244 | 28517530 |
|  |  | M1 | 1528135 | 1000813 | 761050 |
|  |  | M2 | 46675 | 24214 | 21333 |
| Acetyl-aspartate | C6H9NO5 | M0 | 79948558 | 36768572 | 19517013 |
|  |  | M1 | 15048042 | 6253166 | 2684572 |
|  |  | M2 | 194291821 | 66597423 | 33416413 |
|  |  | M3 | 28710358 | 33480216 | 9930634 |
|  |  | M4 | 8435833 | 15983368 | 3263732 |
|  |  | M5 | 63411819 | 71024463 | 18271370 |
|  |  | M6 | 1741848 | 1576994 | 191239 |
| Nicotinamide | C6H6N2O | M0 | 73200305 | 79070526 | 36443691 |
|  |  | M1 | 5299648 | 5559835 | 2544218 |
|  |  | M2 | 1503875 | 3391384 | 1317791 |
|  |  | M3 | 2030478 | 4457120 | 1792313 |
|  |  | M4 | 3927325 | 5021119 | 2441758 |
|  |  | M5 | 13637757 | 25190045 | 12407401 |
|  |  | M6 | 2945011 | 3762648 | 2178516 |
| GABA | C4H9NO2 | M0 | 36009273 | 132813648 | 50872923 |
|  |  | M1 | 840536 | 2863157 | 1321808 |
|  |  | M2 | 808119 | 1177458 | 186245 |
|  |  | M3 | 696343 | 412267 | 802514 |
|  |  | M4 | 7108168 | 3026747 | 3618683 |
| Methionine | C5H11NO2S | M0 | 1681419498 | 1563203405 | 835074013 |
|  |  | M1 | 100700490 | 92740588 | 50285617 |
|  |  | M2 | 10769329 | 9293707 | 4501119 |
|  |  | M3 | 5950353 | 4021868 | 1225149 |
|  |  | M4 | 3746944 | 5997996 | 3095326 |
|  |  | M5 | 13496 | 42858 | 0 |
| Arginine | C6H14N4O2 | M0 | 1524745182 | 1327533669 | 870090222 |
|  |  | M1 | 105886978 | 96071991 | 60372808 |
|  |  | M2 | 61724728 | 57332865 | 28779930 |
|  |  | M3 | 44717528 | 44164220 | 21603303 |
|  |  | M4 | 35254086 | 25160539 | 9926519 |
|  |  | M5 | 9497143 | 6545909 | 3268845 |
|  |  | M6 | 4051551 | 4423627 | 2200570 |
| Citrulline | C6H13N3O3 | M0 | 490470443 | 464905110 | 422828894 |
|  |  | M1 | 36829612 | 36324413 | 33046507 |
|  |  | M2 | 3220748 | 1924963 | 2527278 |
|  |  | M3 | 836456 | 1499103 | 922779 |
|  |  | M4 | 909521 | 2804518 | 1264145 |
|  |  | M5 | 225616 | 394444 | 611405 |
|  |  | M6 | 31614 | 5068 | 4523 |
| Proline | C5H9NO2 | M0 | 7146597568 | 6666628211 | 3529184942 |
|  |  | M1 | 395061308 | 367259142 | 192270079 |
|  |  | M2 | 49216201 | 52161184 | 25295348 |
|  |  | M3 | 0 | 0 | 0 |
|  |  | M4 | 5544554 | 6238298 | 2633540 |
|  |  | M5 | 26273136 | 1748649 | 1271305 |
| Ornithine | C5H12N2O2 | M0 | 157228763 | 310520669 | 119191957 |
|  |  | M1 | 8715147 | 17357622 | 6455147 |
|  |  | M2 | 4227594 | 4470747 | 2599464 |
|  |  | M3 | 1054184 | 1349333 | 449165 |
|  |  | M4 | 1405551 | 5216437 | 2470275 |
|  |  | M5 | 1405551 | 5214683 | 2470275 |
| Acetyl-ornithine | C7H14N2O3 | M0 | 88788469 | 116494913 | 49881897 |
|  |  | M1 | 10101169 | 17822424 | 7492363 |
|  |  | M2 | 37709376 | 99568715 | 56041417 |
|  |  | M3 | 6435500 | 10509718 | 6932653 |
|  |  | M4 | 12385891 | 22651441 | 12939029 |
|  |  | M5 | 5976031 | 7479674 | 6446909 |
|  |  | M6 | 4342116 | 15212027 | 7186507 |
|  |  | M7 | 601765 | 906864 | 1014155 |
| Glutamate | C5H9NO4 | M0 | 429105477 | 498045817 | 201974370 |
|  |  | M1 | 23891932 | 22150420 | 12043143 |
|  |  | M2 | 18473490 | 40096293 | 16143741 |
|  |  | M3 | 4632820 | 13408837 | 2569034 |
|  |  | M4 | 13263661 | 44606887 | 15054705 |
|  |  | M5 | 8817478 | 8697953 | 4777772 |
| Valine | C5H11NO2 | M0 | 4246358214 | 3258297558 | 1433522901 |
|  |  | M1 | 255537649 | 201562760 | 88510981 |
|  |  | M2 | 23071593 | 16962306 | 7729998 |
|  |  | M3 | 440473 | 405676 | 127993 |
|  |  | M4 | 1816864 | 222204 | 257744 |
|  |  | M5 | 16513903 | 601725 | 1096835 |
| Leucine/isoleucine | C6H13NO2 | M0 | 2322748556 | 3182522278 | 1193888351 |
|  |  | M1 | 99321595 | 92313931 | 52620720 |
|  |  | M2 | 10930336 | 11096370 | 5449009 |
|  |  | M3 | 852157 | 597129 | 188109 |
|  |  | M4 | 468323 | 1128004 | 98724 |
|  |  | M5 | 1138842 | 1403350 | 309835 |
|  |  | M6 | 2538013 | 3782335 | 1140201 |
| Alanine | C3H7NO2 | M0 | 725802487 | 696804328 | 332049512 |
|  |  | M1 | 34575817 | 34504224 | 15139653 |
|  |  | M2 | 53445663 | 32451595 | 8946587 |
|  |  | M3 | 78338457 | 12291802 | 7323862 |
| Threonine | C4H9NO3 | M0 | 456362193 | 650097355 | 266915746 |
|  |  | M1 | 25095098 | 34594210 | 13715196 |
|  |  | M2 | 3729974 | 5169693 | 1704340 |
|  |  | M3 | 1975704 | 2874868 | 560131 |
|  |  | M4 | 116282 | 190129 | 34014 |
| Asparagine | C4H8N2O3 | M0 | 17866228 | 19749670 | 19228046 |
|  |  | M1 | 4747260 | 9557208 | 4226422 |
|  |  | M2 | 2157632 | 2710459 | 1449543 |
|  |  | M3 | 15855246 | 4704996 | 11663923 |
|  |  | M4 | 2925516 | 282328 | 3610222 |
| Aspartate | C4H7NO4 | M0 | 235690903 | 423949432 | 83115981 |
|  |  | M1 | 19511177 | 27709211 | 5071997 |
|  |  | M2 | 24334388 | 63390446 | 7456021 |
|  |  | M3 | 185822952 | 264537895 | 56417194 |
|  |  | M4 | 13913494 | 15566020 | 3048970 |
| MTA | C11H15O3N5S | M0 | 16142683 | 6465154 | 7767828 |
|  |  | M1 | 1823634 | 285227 | 205295 |
|  |  | M2 | 2590974 | 402954 | 640231 |
|  |  | M3 | 2337180 | 376573 | 461847 |
|  |  | M4 | 2734913 | 275874 | 521931 |
|  |  | M5 | 10885563 | 1583915 | 2335082 |
|  |  | M6 | 2097658 | 1241876 | 958122 |
| SAM | C15H23O5N6S | M0 | 1561670 | 4811016 | 3460995 |
|  |  | M1 | 93332 | 840014 | 477273 |
|  |  | M2 | 16785 | 29735 | 21273 |
|  |  | M3 | 6073 | 11657 | 4730 |
|  |  | M4 | 12562 | 24531 | 13334 |
|  |  | M5 | 786065 | 945292 | 516042 |
|  |  | M6 | 33630 | 644828 | 183567 |
|  |  | M7 | 4743 | 1002211 | 539080 |
|  |  | M8 | 0 | 247512 | 55924 |
|  |  | M9 | 6184 | 79087 | 3532 |
| N-Acetylputrescine | C6H14ON2 | M0 | 160761520 | 46133842 | 22849768 |
|  |  | M1 | 22190931 | 5052594 | 2413122 |
|  |  | M2 | 184465448 | 20535070 | 15180450 |
|  |  | M3 | 10341831 | 897007 | 567669 |
|  |  | M4 | 9069702 | 1063345 | 686065 |
|  |  | M5 | 394778 | 0 | 0 |
|  |  | M6 | 9883060 | 920862 | 901852 |
| Tyrosine | C9H11NO3 | M0 | 473611103 | 573847078 | 186813459 |
|  |  | M1 | 44773351 | 57256003 | 17202637 |
|  |  | M2 | 1691561 | 1877658 | 548968 |
|  |  | M3 | 10691 | 9447 | 7053 |
|  |  | M4 | 8117 | 10542 | 4383 |
|  |  | M5 | 15237 | 11543 | 0 |
|  |  | M6 | 290964 | 55043 | 17871 |
|  |  | M7 | 514590 | 59045 | 0 |
|  |  | M8 | 1899796 | 1824521 | 969717 |
|  |  | M9 | 3010953 | 2411780 | 712117 |
| Guanosine | C10H13N5O5 | M0 | 23346917 | 20919784 | 8547182 |
|  |  | M1 | 2477223 | 1968694 | 674786 |
|  |  | M2 | 631783 | 149996 | 37471 |
|  |  | M3 | 318466 | 7831 | 4386 |
|  |  | M4 | 471747 | 8284 | 4498 |
|  |  | M5 | 2712888 | 183916 | 195455 |
|  |  | M6 | 80093 | 20197 | 4254 |
|  |  | M7 | 446567 | 750526 | 22645 |
|  |  | M8 | 0 | 8487 | 3681 |
|  |  | M9 | 0 | 14271 | 0 |
|  |  | M10 | 3684 | 8530 | 2906 |

Table S12. HILIC-HRMS-ESI(‒) peak areas of metabolites in the cellular fraction of the human fecal microbiome incubated with [U-^13^C]-inulin.

| Metabolite | Formula | Isotopologue | Polar_neg1 | Polar_neg2 | Polar_neg3 |
| --- | --- | --- | --- | --- | --- |
| Succinate | C4H6O4 | M0 | 165125599 | 30833994 | 24574420 |
|  |  | M1 | 20246737 | 3439976 | 917363 |
|  |  | M2 | 26754168 | 8112379 | 3267162 |
|  |  | M3 | 10213076 | 3276667 | 606818 |
|  |  | M4 | 370045 | 264126 | 15540 |
| Fumarate | C4H4O4 | M0 | 13014015 | 7819397 | 4945859 |
|  |  | M1 | 1234769 | 470939 | 285395 |
|  |  | M2 | 908634 | 523886 | 53218 |
|  |  | M3 | 2740456 | 1030336 | 523056 |
|  |  | M4 | 76358 | 3396 | 4344 |
| Malate | C4H6O5 | M0 | 204043216 | 124822987 | 93272938 |
|  |  | M1 | 20641699 | 9331105 | 6984457 |
|  |  | M2 | 18956888 | 7990657 | 3578700 |
|  |  | M3 | 58612280 | 5828914 | 10383996 |
|  |  | M4 | 4461632 | 194370 | 390369 |
| Lactate | C3H6O3 | M0 | 177688618 | 240151633 | 97090920 |
|  |  | M1 | 6837790 | 7699265 | 2735453 |
|  |  | M2 | 12511279 | 2797262 | 1019032 |
|  |  | M3 | 19500117 | 623079 | 921042 |
| Glucose 6-phosphate | C6H13O9P | M0 | 6674245 | 2413476 | 1097072 |
|  |  | M1 | 671258 | 172948 | 83597 |
|  |  | M2 | 527985 | 125391 | 23592 |
|  |  | M3 | 68273 | 6476 | 3897 |
|  |  | M4 | 991208 | 3988 | 2863 |
|  |  | M5 | 1066000 | 2161 | 2907 |
|  |  | M6 | 4749238 | 487357 | 161894 |
| Phosphoenolpyruvate | C3H5O6P | M0 | 6561167 | 8343523 | 688619 |
|  |  | M1 | 11180 | 91020 | 5691 |
|  |  | M2 | 0 | 0 | 6675 |
|  |  | M3 | 2963031 | 1686752 | 429453 |
| Butyrate | C4H9O2 | M0 | 38815014 | 149700163 | 16806013 |
|  |  | M1 | 2315013 | 12989214 | 880153 |
|  |  | M2 | 5249 | 8022 | 2922 |
|  |  | M3 | 299925 | 11352057 | 258995 |
|  |  | M4 | 5082904 | 73605919 | 3156593 |
| Pyruvate | C3H4O3 | M0 | 178582203 | 94703294 | 89466624 |
|  |  | M1 | 24425 | 5072 | 0 |
|  |  | M2 | 5697781 | 2919400 | 3062742 |
|  |  | M3 | 8746154 | 2140510 | 1861897 |
| Propionate | C3H6O2 | M0 | 108924093 | 208462723 | 34161659 |
|  |  | M1 | 12328370 | 22002953 | 2829461 |
|  |  | M2 | 46951505 | 175347664 | 14646535 |
|  |  | M3 | 12107872 | 49635767 | 5129832 |
| Alpha-ketoglutarate | C5H6O5 | M0 | 19183489 | 12604789 | 11428239 |
|  |  | M1 | 867316 | 790484 | 309397 |
|  |  | M2 | 423275 | 1362983 | 397608 |
|  |  | M3 | 83106 | 263046 | 19494 |
|  |  | M4 | 586144 | 1407748 | 627769 |
|  |  | M5 | 2647128 | 1129181 | 597603 |
| 3-Phosphoglyceric acid | C3H7O7P | M0 | 6301690 | 5542161 | 2747751 |
|  |  | M1 | 157228 | 81830 | 3881 |
|  |  | M2 | 5709 | 6896 | 4044 |
|  |  | M3 | 590247 | 29204 | 9753 |
| Hydroxyglutarate | C5H8O5 | M0 | 261812500 | 135690655 | 94759182 |
|  |  | M1 | 15955499 | 8111365 | 5362448 |
|  |  | M2 | 8514421 | 4390255 | 3005597 |
|  |  | M3 | 2647128 | 1129181 | 642026 |
|  |  | M4 | 4497635 | 2192834 | 1673958 |
|  |  | M5 | 6933611 | 1751338 | 2035482 |

Table S13. LC-HRMS peak areas of polyamines and related metabolites in the cellular fraction of *Bacteroides* incubated with [U-^13^C]-inulin.

| Metabolite | Formula | Fragment | B. fragilis_1 | B. fragilis_2 | B. fragilis_3 | B. thetaiotaomicron_1 | B. thetaiotaomicron_2 | B. thetaiotaomicron_3 |
| --- | --- | --- | --- | --- | --- | --- | --- | --- |
| [Fmoc]3-SPD | C52H49N3O6 | M0 | 118361750 | 84898714 | 89880428 | 190503850 | 163191738 | 126927154 |
|  |  | M1 | 64113324 | 42593391 | 46495818 | 109921630 | 94413605 | 73751166 |
|  |  | M2 | 21957379 | 15099114 | 16775906 | 40600075 | 35859934 | 28295728 |
|  |  | M3 | 3973233 | 3414892 | 2651411 | 12424523 | 10595070 | 8503437 |
|  |  | M4 | 323803 | 206742 | 279530 | 2206450 | 1917533 | 2030156 |
|  |  | M5 | 0 | 0 | 0 | 0 | 95608 | 180575 |
|  |  | M6 | 0 | 0 | 0 | 0 | 0 | 0 |
|  |  | M7 | 0 | 0 | 0 | 0 | 0 | 0 |
| [Fmoc]2-PUT | C34H32N2O4 | M0 | 6223224996 | 5965679810 | 5917271463 | 8252999393 | 7921684488 | 7654749238 |
|  |  | M1 | 2354888393 | 2261418435 | 2234114882 | 3137466614 | 3010338082 | 2920313795 |
|  |  | M2 | 471489381 | 341036283 | 400291367 | 615813691 | 539821575 | 572755383 |
|  |  | M3 | 67940912 | 66581239 | 64309697 | 88894722 | 88236863 | 85443182 |
|  |  | M4 | 20974902 | 20953929 | 20556235 | 25381672 | 23918013 | 24054386 |
| [Fmoc]2-ornithine | C35H32N2O6 | M0 | 42002743 | 29957593 | 42539508 | 25127289 | 21793979 | 20837718 |
|  |  | M1 | 16015109 | 11392830 | 16329269 | 9259931 | 8222380 | 8220533 |
|  |  | M2 | 2386285 | 1631807 | 2716156 | 944348 | 973768 | 1220313 |
|  |  | M3 | 291518 | 99909 | 201934 | 65385 | 41749 | 27409 |
|  |  | M4 | 0 | 0 | 0 | 0 | 0 | 0 |
|  |  | M5 | 31589 | 0 | 0 | 0 | 36650 | 0 |
| [Fmoc]1-citrulline | C21H23N3O5 | M0 | 386824102 | 341746017 | 419375054 | 386756017 | 371186548 | 329013160 |
|  |  | M1 | 90138448 | 80527794 | 97598920 | 91316082 | 88310187 | 77382027 |
|  |  | M2 | 12767660 | 11036345 | 13481494 | 12381168 | 12346710 | 10756764 |
|  |  | M3 | 182925 | 126307 | 350961 | 349733 | 283350 | 308548 |
|  |  | M4 | 0 | 0 | 0 | 0 | 0 | 0 |
|  |  | M5 | 0 | 0 | 55186 | 32670 | 31893 | 0 |
|  |  | M6 | 75827 | 113484 | 67093 | 56428 | 93991 | 0 |
| [Fmoc]1-arginine | C21H24N4O4 | M0 | 10584387 | 7478274 | 10304208 | 25749113 | 23333607 | 19919842 |
|  |  | M1 | 1211361 | 905522 | 872928 | 2121476 | 2824611 | 1518817 |
|  |  | M2 | 190684 | 104933 | 28580 | 79021 | 306918 | 199734 |
|  |  | M3 | 0 | 0 | 0 | 0 | 0 | 0 |
|  |  | M4 | 44706 | 0 | 0 | 0 | 0 | 0 |
|  |  | M5 | 45409 | 123560 | 41621 | 33883 | 36518 | 51022 |
|  |  | M6 | 46975 | 37207 | 0 | 195933 | 50416 | 131383 |
| [Fmoc]1-agmatine | C20H24N4O2 | M0 | 445385289 | 382774891 | 393287975 | 133515883 | 129372670 | 119345892 |
|  |  | M1 | 76224370 | 67370003 | 69141037 | 20387666 | 18991318 | 18258429 |
|  |  | M2 | 9319660 | 8060562 | 7943166 | 2141543 | 2067463 | 2304815 |
|  |  | M3 | 340568 | 37131 | 92983 | 76914 | 0 | 50926 |
|  |  | M4 | 29258 | 88665 | 0 | 60156 | 0 | 0 |
|  |  | M5 | 0 | 0 | 0 | 0 | 34932 | 39010 |
| [Fmoc]1-carbamoylputrescine | C20H23N3O3 | M0 | 386371569 | 356582408 | 381219916 | 120163697 | 120187483 | 112514162 |
|  |  | M1 | 83026011 | 76737723 | 81821497 | 26073758 | 25606983 | 24402015 |
|  |  | M2 | 8215330 | 8020190 | 8185060 | 2409937 | 2260889 | 2144591 |
|  |  | M3 | 218158 | 100733 | 235509 | 76019 | 75029 | 535756 |
|  |  | M4 | 0 | 0 | 0 | 0 | 0 | 0 |
|  |  | M5 | 332849 | 225525 | 121381 | 63741 | 198669 | 212983 |
| [Fmoc]1-acetylputrescine | C21H24N2O3 | M0 | 7166858037 | 6868102453 | 7039969273 | 4065694984 | 4170268080 | 4006532270 |
|  |  | M1 | 1728087002 | 1674650616 | 1708020483 | 976271192 | 1005567808 | 961202163 |
|  |  | M2 | 877240234 | 834263422 | 867374526 | 586922534 | 597182387 | 573836403 |
|  |  | M3 | 148949617 | 145314087 | 147523675 | 99667791 | 102946662 | 99134107 |
|  |  | M4 | 29946600 | 27225129 | 29415459 | 17498419 | 15623425 | 15175061 |
|  |  | M5 | 1505232 | 1040183 | 1834715 | 0 | 675617 | 205606 |
|  |  | M6 | 343785 | 316991 | 0 | 0 | 0 | 0 |
| [Fmoc]1-diacetylspermidine | C26H33N3O4 | M0 | 66066679 | 61473501 | 62227545 | 4155679 | 4038410 | 3711667 |
|  |  | M1 | 18602560 | 16335644 | 17704327 | 332814 | 472902 | 480663 |
|  |  | M2 | 8585418 | 7289909 | 7878212 | 593990 | 310486 | 558709 |
|  |  | M3 | 1228573 | 1117251 | 1419896 | 0 | 0 | 0 |
|  |  | M4 | 56073 | 0 | 0 | 49217 | 86680 | 63233 |
|  |  | M5 | 0 | 0 | 0 | 0 | 0 | 0 |
|  |  | M6 | 454166 | 367388 | 314552 | 1266457 | 298705 | 428749 |
|  |  | M7 | 0 | 0 | 0 | 0 | 0 | 0 |
|  |  | M8 | 0 | 0 | 0 | 0 | 0 | 61670 |
|  |  | M9 | 0 | 0 | 0 | 0 | 0 | 0 |
|  |  | M10 | 37858 | 0 | 0 | 35218 | 183880 | 31417 |
|  |  | M11 | 0 | 0 | 0 | 0 | 0 | 0 |
| MTA | C11H15O3N5S | M0 | 17142134 | 22400428 | 26972911 | 16823445 | 15249710 | 17783618 |
|  |  | M1 | 814969 | 468295 | 1270136 | 382423 | 247646 | 1023037 |
|  |  | M2 | 434499 | 342984 | 341876 | 241146 | 107360 | 72227 |
|  |  | M3 | 0 | 0 | 0 | 0 | 0 | 0 |
|  |  | M4 | 0 | 0 | 0 | 0 | 35488 | 0 |
|  |  | M5 | 35233 | 31017 | 32594 | 48355 | 50634 | 48017 |
|  |  | M6 | 0 | 0 | 0 | 0 | 0 | 0 |
| SAM | C15H23N6O5S | M0 | 14930166 | 23036069 | 20744881 | 17700394 | 20109981 | 16030621 |
|  |  | M1 | 2366517 | 3836360 | 3359302 | 2452176 | 3177340 | 2312110 |
|  |  | M2 | 0 | 98188 | 104029 | 0 | 49488 | 33122 |
|  |  | M3 | 14398 | 0 | 0 | 17664 | 0 | 0 |
|  |  | M4 | 0 | 0 | 0 | 0 | 0 | 0 |
|  |  | M5 | 108672 | 35334 | 153909 | 92242 | 35985 | 209843 |
|  |  | M6 | 146068 | 82971 | 85843 | 69702 | 69424 | 78733 |
|  |  | M7 | 0 | 0 | 0 | 0 | 0 | 0 |
|  |  | M8 | 16532 | 17575 | 19702 | 15945 | 22735 | 14668 |
|  |  | M9 | 0 | 0 | 0 | 0 | 0 | 0 |


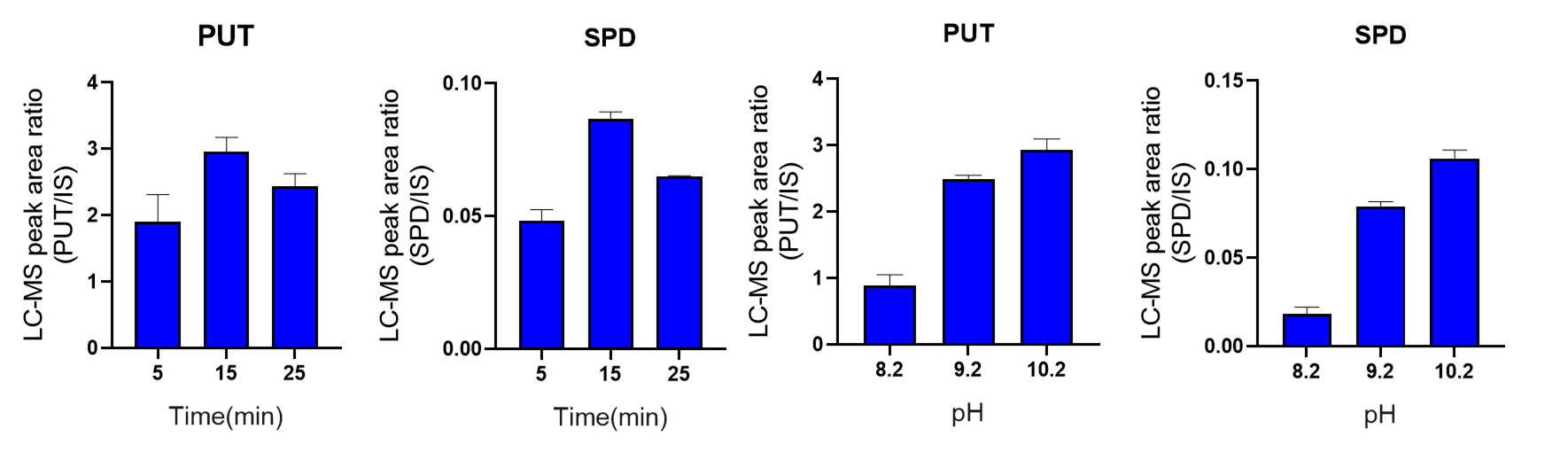


Figure S1. Optimization of the reaction time and pH of the Fmoc-OSu derivatization reaction for the analysis of PUT and SPD. The samples were detected by LC-MS/MS, and the ratios of the peak area of the analyte to that of tolbutamide (internal standard) were compared and reported. The optimal conditions were obtained with a reaction time of 15 minutes and a pH at 10.2. Values shown are mean ± SEM (n = 3).

Figure S2. ^13^C fractional enrichments of polar metabolites in the human fecal microbiome. Fresh fecal microbes were incubated with [U-^13^C]-inulin anaerobically for 24 h. Samples were processed and analyzed by HILIC-HRMS as described in the Methods. [U-^13^C]-inulin carbons were traced through **a**) Central carbon metabolism pathway and **b-c**) Amino acid metabolism pathway. The x-axis denotes the number of ^13^C atoms present in each compound, and the y-axis indicates the ^13^C enrichment. Values shown are mean ± SEM (n = 3).

Figure S3. ^13^C-labeled fraction of polyamine and related metabolites in the microbial cell fraction and the culture medium for (a) human fecal microbiome samples and (b) mouse fecal microbiome samples. The left side of each panel represents the metabolites detected in the culture media (orange bar), and the right side of each panel represents the metabolites detected in the microbial cells (blue bar). The metabolites are listed in the descending order of the ^13^C fraction in the microbial cells, with the highest labeled metabolite listed at the top of the graph. Values shown are mean ± SEM (n = 3). *p < 0.05; **p < 0.01; and ***p < 0.001, as indicated.

**b**

**a**

Figure S4. Analysis of polyamine levels across different disease states using fecal calprotectin as a biomarker. Data were obtained from the public available fecal metabolomic data of IBD patients (https://ibdmdb.org/). (a) Ulcerative colitis patients; (b) Crohn’s disease patients. X-axis: Fecal calprotectin (μg/g); Y-axis: LC-MS peak area of polyamines (* p < 0.05).

**
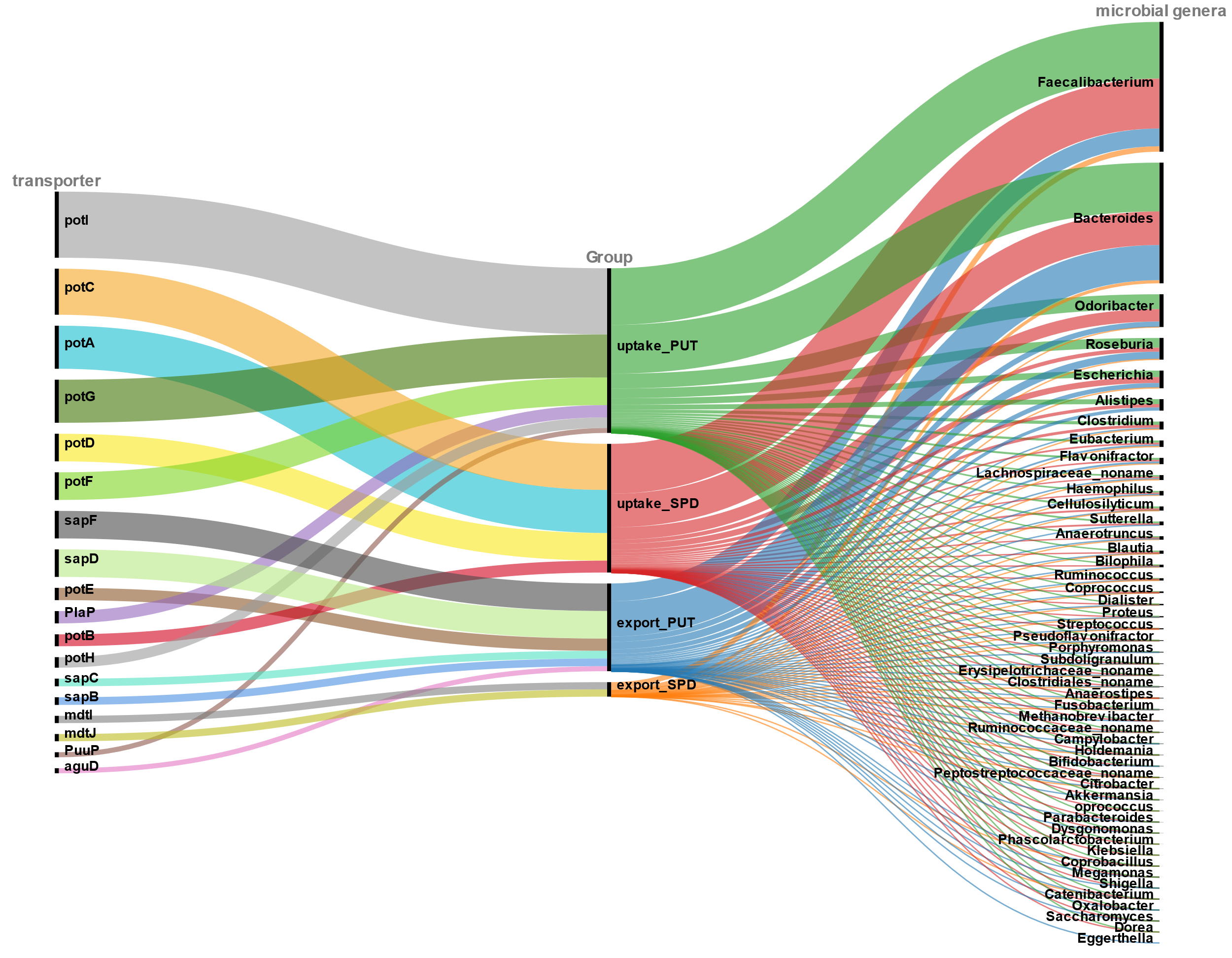
**

Figure S5. Sankey diagram of the connections between polyamine transporters and gut microbiome genera. Metatranscriptomic data from the Inflammatory Bowel Disease Multi-omics Database (IBDMDB, https://ibdmdb.org/) were analyzed to show the connections between polyamine transporters and gut microbiome genera. *Faecalibacterium* and *Bacteroides* were identified as the major bacterial genera expressing polyamine transporters.

Figure S6. Time-dependent changes of PUT and SPD levels in the culture medium of *Bacteroides* spp. Samples were analyzed by LC-MS/MS, and values shown are mean ± SEM (n = 3) in nM.

Figure S7. Maximum-likelihood phylogenetic tree of the human gut microbiome in polyamine biosynthesis. TBLASTN was used to identify microbial species with genes encoding polyamine metabolism enzymes in the NCBI RefSeq Representative Genome Database and speA was used as a query. Bootstrap values are indicated by shaded circles at each node. *Bacteroides* spp. have minimal species variation in speA gene, indicating the significance of *Bacteroides* in SPD biosynthesis.
